# Association Between New-Onset Medicaid Home Care and Family Caregivers’ Health

**DOI:** 10.1001/jamahealthforum.2021.2671

**Published:** 2021-09-17

**Authors:** Emily S. Unger, David C. Grabowski, Jarvis T. Chen, Lisa F. Berkman

**Affiliations:** 1 Harvard Medical School, Boston, Massachusetts; 2 Harvard T.H. Chan School of Public Health, Boston, Massachusetts; 3 Department of Health Care Policy, Harvard Medical School, Boston, Massachusetts

## Abstract

**Importance:**

More than 17 million people in the US provide uncompensated care for adults with physical or cognitive limitations. Such caregiving is associated with worse mental and physical health, yet little research has investigated how publicly funded home care might ameliorate these harms.

**Objective:**

To investigate the association between Medicaid home care services and family caregivers’ health.

**Design, Setting, and Participants:**

This longitudinal cohort study used data from the 1996 to 2017 Medical Expenditures Panel Survey. Data on all household members were collected in 5 interviews over 2 years. Person-level difference-in-difference models were used to isolate within-person changes associated with new onset of Medicaid home care. The Medical Expenditures Panel Survey longitudinal data sets included 331 202 individuals (approximately 10% excluded owing to loss to follow-up). Adult (age ≥21 years) members of households that contained at least 1 person with limited activities of daily living were included in our study. The analysis itself was performed from March to August of 2020.

**Exposures:**

New onset of regular (≥1 time per month) Medicaid home care in the household.

**Main Outcomes and Measures:**

Self-rated mental and physical health (planned prior to beginning the study).

**Results:**

The study population was 14 013 adults; 7232 were “likely caregivers,” or nondisabled adult coresidents of someone with activities of daily living limitations. Overall, 962 likely caregivers were ever exposed to Medicaid home care in the household; for 563, we observed the onset. Of likely caregivers exposed to Medicaid home care, 479 (50%) were women; 296 (31%) were White non-Hispanic, 309 (31%) were Hispanic or Latinx, and 279 (29%) were Black non-Hispanic individuals, respectively; 326 (34%) had less than a high school education; and 300 (31%) were in or near poverty. Median age of participants was 51 (interquartile range, 39-62) years. New-onset Medicaid home care was associated with a 0.08 standard deviation improvement in likely caregivers’ self-rated mental health (95% CI, 0.01-0.14; *P* = .02) measured 1 to 6 months after onset, equivalent to a 3.39% improvement (95% CI, 0.05%-6.33%) over their average preonset mental health. No association with self-rated physical health was found (<0.001 standard deviations; 95% CI, −0.06 to 0.06; *P* = .99).

**Conclusions and Relevance:**

In this cohort study, Medicaid home care was associated with improvement in caregiver self-rated mental health, but not with any short-term change in self-rated physical health. When evaluating the social value of home care programs, policy makers should consider spillover benefits to caregivers.

## Key Points

**Question:**

How does family caregivers’ health change when the person they care for begins to receive Medicaid home care services?

**Findings:**

In this longitudinal cohort study, family caregivers’ self-rated mental health improved significantly after their family member began receiving Medicaid home care services. Their self-rated physical health did not change.

**Meaning:**

The findings of this longitudinal cohort study suggest that Medicaid home care programs may have spillover benefits, affecting not only their direct recipients but also those recipients’ family caregivers.

## Teaser

This cohort study examines the association between Medicaid home care services and family caregivers’ health using data from the 1996 to 2017 Medical Expenditures Panel Survey.

## Introduction

In the US, more than 17 million people care for family or friends who need assistance with activities of daily living.^1^ Like most domestic and care labor, this labor is often unpaid, invisible, and performed by women.^2,3,4^ When paid, caregiving labor is largely performed by immigrant women and women of color, paid below a living wage, and excluded from labor protections.^5,6,7^ Epidemiology, economics, and psychology research shows that uncompensated caregiving is associated with worse physical and mental health, including depression and anxiety^2,8,9,10,11,12,13,14,15,16,17^; elevated cardiometabolic risk factors^11,18,19,20,21^; and higher rates of future cardiovascular disease,^12,18,22,23^ frailty,^24^ and, according to some studies, death.^25,26,27,28,29,30,31,32^ These harms are concentrated among women, people with low incomes, people providing more intensive care, and people without choice in becoming caregivers.^8,18,19,33^ Although caregiving can provide a sense of purpose and connection, in the US, these benefits are typically offset by physical, financial, and emotional strain.^1,11,28,34^

Public policy plays a role in structuring caregiving and can mitigate or exacerbate its associated health harms. Research has shown that policies supporting caregivers of children, like paid parental leave and publicly funded childcare, benefit these caregivers’ health.^35,36,37,38^ Similarly, respite care, payments to family caregivers,^39^ and case management have been shown to improve the well-being of caregivers for adults.^40,41,42,43,44,45,46,47,48,49^ Yet although caregiving-associated health harms are structural—shaped by policies, social norms, and economic pressures grounded in sexism, ableism, racism, and classism^50,51,52,53,54^—many efforts to ameliorate them focus on individual-level interventions like support groups and education.^1,3,55,56,57,58^ Though these interventions may help caregivers cope, they do not change the demands of caregiving or address the underlying social and economic pressures.

Over recent decades, US long-term care policy has undergone a major shift toward home- and community-based services.^59,60^ Still, access to these services remains limited and varies widely from state to state.^61,62,63^ To investigate how these structural factors shape caregivers’ health, we used an ecosocial theoretical framework,^64^ longitudinal data from the Medical Expenditures Panel Survey (MEPS), and individual-level difference-in-difference models to assess the relationship between onset of Medicaid home care and family caregivers’ health. Only Medicaid—the means-tested public health insurance program—provides long-term home care services in the US; Medicare, the universal health insurance program for people aged 65 years or older or with disabilities, provides just limited, short-term services.^59,62,65,66,67^ We focused on publicly funded long-term care, rather than private-paid care, because such programs can reduce caregivers’ physical and emotional burden without increasing their financial burden.^65,68,69,70,71^ Little research has investigated how publicly funded home care affects caregivers, and existing studies do not assess caregiver health outcomes, are from outside the US, or compare home care to institutionalization rather than to continued stand-alone family caregiving.^72,73,74,75,76,77^

We hypothesized that family caregivers’ health would improve when their care recipient begins receiving Medicaid home care, and that these benefits would vary by gender, race or ethnicity, socioeconomic status, and age of the caregiver, and by care recipient cognitive impairment.^8,19,71,78,79,80,81^ If such spillover exists, this would have important policy implications: investment in Medicaid home care might produce greater population health gains than previously described, providing benefits not traditionally counted in cost-benefit analyses.

## Methods

### Setting, Data Sources, Participants, and Study Size

The MEPS is a nationally representative, longitudinal survey with data on households’ health care utilization and spending. Recruitment and data collection methods are described elsewhere.^82^ All data were deidentified and publicly available, and our study was deemed not human participants research by the Harvard University institutional review board. We followed the Strengthening the Reporting of Observational Studies in Epidemiology (STROBE) reporting guidelines.^83^

We used data from all 21 complete MEPS panels available at the time of analysis (March-August 2020), which span the years 1996 to 2017. Each household was interviewed 5 times: once every 6 months for 2 years. We used MEPS longitudinal data files, which exclude individuals lost to follow-up (approximately 10%; exact share varies by panel). We limited our sample to households that included an adult with activities of daily living (ADL) limitations, assessed by a single question about “help or supervision with personal care such as bathing, dressing, or getting around the house because of an impairment or a physical or mental health problem.” We excluded children younger than 21 years and individuals who lived in 2 or more households during the survey, lived alone, or were missing covariates or outcomes. Further details are available in eMethods: Population in the Supplement.

### Study Design and Approach to Bias

Only people with low income and assets and medical need are eligible for Medicaid home care; these factors are associated with worse health among other household members. Thus, rather than comparing households that did and did not receive Medicaid home care—which would be biased by unobserved confounding—we used person-level difference-in-difference models to estimate within-person change in outcomes associated with within-person onset of the exposure.^84,85^ Models were conditional on person and round, controlling for all person-level, time-invariant confounding and overall time trends in the outcomes. To control time-varying confounding, we included person- and household-level covariates.

### Variables and Measurement

The primary exposure was new onset of regular Medicaid home care. At each interview, a household respondent reported all health services received by household members since the last interview; the payer was documented in this interview and by contacting the relevant physicians, hospitals, home health agencies, and/or pharmacies. We defined regular Medicaid home care as any home health services paid by Medicaid and received more than once per month for at least 1 month during the reference period. If anyone received Medicaid home care in a given round, everyone in the household was considered exposed (eMethods: Variables in the Supplement).

To distinguish direct effects of Medicaid home care on recipients from potential spillover effects on caregivers, we categorized household members as either Medicaid home care recipients, disabled nonrecipients (adults with ADL limitations who never received Medicaid home care), or likely caregivers (adult nonrecipients without ADL limitations). People changed exposure status frequently (eFigures 1 and 2 in the Supplement), so our primary exposure was initial onset of Medicaid home care because this was straightforward to operationalize and substantively important. We defined an exposure variable that was 0 in rounds with no Medicaid home care and 1 in the first round with Medicaid home care in the household; after that round, individuals dropped out of the model. Thus, our estimates reflect within-person changes in the outcome associated with the initial onset of Medicaid home care, above and beyond any changes in health experienced by unexposed individuals over time.

The primary outcomes were self-rated physical and mental health. Household respondents reported both measures on a scale from 1 (excellent) to 5 (poor). We standardized these scales and reversed the coding so that positive associations indicate improvement in self-rated mental or physical health. Outcomes were measured at each interview, ensuring that exposures temporally preceded outcomes.

Person-level covariates included hospitalizations and emergency department visits between the previous and current interviews (1, 2, 3, 4, or ≥5), number of nights spent in hospital between the previous and current interviews, and employment status at the interview date (employed vs unemployed). Household-level covariates included the number of other household members with fair/poor self-rated physical and mental health (1, 2, 3, 4, or ≥5) at the interview date, whether any other household members were hospitalized or had emergency department visits since the last interview, and whether anyone in the household was employed at the interview date. We selected these covariates because they were available in every round and because socioeconomic status, prior health status, and family members’ health status are known determinants of both Medicaid eligibility and current health status (eMethods: Variables in the Supplement).

We assessed 6 potential association measure modifiers: gender (women vs men), baseline poverty status (in or near poverty vs low/middle/high income), whether someone in the household had a cognitive limitation/dementia (yes vs no), employment status (employed vs unemployed), age (younger than 65 years vs older than 65 years), and race and ethnicity (individuals identifying as non-Hispanic White, non-Hispanic Black, Hispanic/Latinx, non-Hispanic Asian, or multiple race/other). These social categories have all been found, in prior research, to be associated with caregiving, degree of caregiver burden, or severity of caregiving-associated health harms.^8,19,71,78,79,86^ All variables were reported by the household respondent using the categories available on the survey instrument.

### Statistical Methods

For descriptive analyses, we used χ^2^ tests. For our primary difference-in-difference analysis, we ran unadjusted models (conditional on person and round), models adjusted for individual-level covariates, and models adjusted for individual- and household-level covariates (fully adjusted). In all models, we included an interaction term between the difference-in-difference variable and household role (likely caregiver, disabled nonrecipient, and Medicaid home care recipient); our primary results are those for the likely caregiver group.

As exploratory secondary analyses, we ran fully adjusted event study models to assess changes in self-rated health in each round before and after the onset of Medicaid home care.^87^ We applied Wald tests to the parameters from these models to test for parallel pretrends, checking for any trend in self-rated health prior to Medicaid home care onset that might render our primary results invalid. In addition, to explore the possibility of association measure modification, we ran separate difference-in-difference-in-difference models for the 6 potential modifiers. In each model, we included a 3-way interaction between the difference-in-difference variable, household role, and demographic variable of interest. Although these models were not fully stratified, we refer to them as “stratified analyses” in the Results section for simplicity. More details, including estimating equations, are in eMethods: Model in the Supplement.

All models used conditional likelihood multivariable linear regression with robust standard errors clustered at the individual level. We did not incorporate the complex survey design because we found that standard errors clustered at the individual level were more conservative (eMethods: Model in the Supplement). We did not apply survey weights because we sought to assess the association between Medicaid home care and self-rated health in households that actually received Medicaid home care in our sample; we have no basis for claiming these results are nationally generalizable. We did perform sensitivity analyses using the survey weights (eMethods: Sensitivity Analyses in the Supplement). We also performed sensitivity analyses comparing self-rated mental health to more clinically interpretable measures of mental health only available in certain rounds (eMethods: Sensitivity Analyses in the Supplement).

All hypothesis tests were 2-sided. For exploratory analyses, we used an a priori statistical significance cut-off of *P* = .05. For our primary analyses, we used a Bonferroni-adjusted cutoff of *P* = .025 to account for testing two primary outcomes (self-rated physical and mental health); because these outcomes are correlated, this approach is conservative. All statistical analysis was conducted using Stata statistical software (version 16, Stata Corp), using the xt series of commands for conditional likelihood models. The analysis itself was performed from March to August of 2020.

## Results

The MEPS longitudinal data sets included 331 202 individuals. After excluding people who lived in 2 or more households (4.2% of the population) and restricting to households where at least 1 adult had ADL limitations, our sample contained 21 184 individuals. We excluded 3713 people who lived alone, 3408 children, and 50 adults who were missing data (demographics available in eTable 1 in the Supplement), for a final sample of 14 013. All these adults contributed to covariate parameter estimates, but because we used conditional likelihood models, only the 1201 living in a household first exposed to Medicaid home care after round 1 contributed to main association estimates. Of these, 560 were Medicaid home care recipients, 563 were likely caregivers, and 78 were disabled nonrecipients. Overall, 30 individuals, mostly Medicaid home care recipients, did not contribute to association estimates because they died or stopped responding after the exposure was measured but before outcomes were measured.

Table 1 and eTables 2 and 3 in the Supplement show descriptive statistics. People living in households where someone received Medicaid home care were more likely than others in the population to be women, unemployed, and Black non-Hispanic or Hispanic or Latinx; they had lower education levels, lower income, and worse self-rated physical and mental health, though similar rates of urgent health care utilization and a similar age distribution were reported. In Medicaid home care-receiving households, the Medicaid home care recipients themselves were more likely to be older and female and have lower education levels and household incomes. They were less likely to be employed and had worse self-rated health and greater urgent health care utilization.

**Table 1.** Demographic and Health Characteristics of Adults in Households With at Least 1 Disabled Adult, 1996 to 2017

Characteristic	Full population (n = 14 013)			Population in households that receive Medicaid home care (n = 2051)			
	No. (%)		*P* value	No. (%)			*P* value
	In household that never receives Medicaid home care (n = 11 962)	In household that receives Medicaid home care (n = 2051)		Likely caregiver (n = 962)	Disabled non-recipient (n = 123)	Medicaid home care recipient (n = 966)	
Age at baseline, median (IQR), y	57.0 (44.0-72.0)	58.0 (44.0-72.0)	.29	51.0 (39.0-62.0)	67.0 (55.0-78.0)	65.0 (50.0-78.0)	<.001
Gender							
Male	5343 (44.7)	844 (41.2)	.003	483 (50.2)	57 (46.3)	304 (31.5)	<.001
Female	6619 (55.3)	1207 (58.8)		479 (49.8)	66 (53.7)	662 (68.5)	
Race and ethnicity							
White non-Hispanic individuals	6325 (52.9)	643 (31.4)	<.001	296 (30.8)	47 (38.2)	300 (31.1)	.22
Hispanic or Latinx individuals	2471 (20.7)	670 (32.7)		309 (32.1)	39 (31.7)	322 (33.3)	
Black non-Hispanic individuals	2260 (18.9)	584 (28.5)		279 (29.0)	31 (25.2)	274 (28.4)	
Asian non-Hispanic individuals	635 (5.3)	102 (5.0)		59 (6.1)	3 (2.4)	40 (4.1)	
Multiple race or other individuals^a^	271 (2.3)	52 (2.5)		19 (2.0)	3 (2.4)	30 (3.1)	
Education level							
Less than or equal to 8th grade	1932 (16.3)	559 (27.6)	<.001	156 (16.4)	45 (36.9)	358 (37.6)	<.001
8th to 12th grade, no diploma	1717 (14.5)	393 (19.4)		170 (17.8)	20 (16.4)	203 (21.3)	
High school diploma or GED	4331 (36.5)	580 (28.6)		321 (33.6)	33 (27.0)	226 (23.8)	
Some college or associate degree	2229 (18.8)	318 (15.7)		184 (19.3)	15 (12.3)	119 (12.5)	
Bachelor’s degree	1099 (9.3)	126 (6.2)		88 (9.2)	5 (4.1)	33 (3.5)	
Graduate school	550 (4.6)	51 (2.5)		35 (3.7)	4 (3.3)	12 (1.3)	
One or more emergency department visits, baseline	990 (8.3)	191 (9.3)	.12	45 (4.7)	10 (8.1)	136 (14.1)	<.001
One or more hospitalizations, baseline	796 (6.7)	163 (7.9)	.03	18 (1.9)	11 (8.9)	134 (13.9)	<.001
Nights in hospital if hospitalized, baseline, median (IQR)	5.0 (2.0-11.0)	6.0 (3.0-12.0)	.09	3.0 (2.0-5.0)	7.0 (3.0-8.0)	7.0 (3.0-15.0)	<.001
Employed at baseline	3902 (32.6)	510 (24.9)	<.001	460 (47.8)	15 (12.2)	35 (3.6)	<.001
In or near poverty at baseline	2935 (24.5)	816 (39.8)	<.001	300 (31.2)	61 (49.6)	455 (47.1)	<.001
Individual ever has cognitive limitations	4069 (34.0)	912 (44.5)	<.001	111 (11.5)	74 (60.2)	727 (75.3)	<.001
Someone else in the household ever has cognitive limitations	5214 (43.6)	1123 (54.8)	<.001	771 (80.1)	88 (71.5)	264 (27.3)	<.001
Self-reported physical health at baseline							
Excellent	1614 (13.5)	224 (10.9)	<.001	181 (18.8)	7 (5.7)	36 (3.7)	<.001
Very good	2300 (19.2)	284 (13.8)		191 (19.9)	9 (7.3)	84 (8.7)	
Good	3199 (26.7)	504 (24.6)		313 (32.5)	25 (20.3)	166 (17.2)	
Fair	2732 (22.8)	583 (28.4)		208 (21.6)	44 (35.8)	331 (34.3)	
Poor	2117 (17.7)	456 (22.2)		69 (7.2)	38 (30.9)	349 (36.1)	
Self-reported mental health at baseline							
Excellent	3010 (25.2)	433 (21.1)	<.001	297 (30.9)	20 (16.3)	116 (12.0)	<.001
Very good	2795 (23.4)	371 (18.1)		234 (24.3)	12 (9.8)	125 (12.9)	
Good	3625 (30.3)	651 (31.7)		308 (32.0)	44 (35.8)	299 (31.0)	
Fair	1685 (14.1)	396 (19.3)		99 (10.3)	30 (24.4)	267 (27.6)	
Poor	847 (7.1)	200 (9.8)		24 (2.5)	17 (13.8)	159 (16.5)	
First round when someone in household receives Medicaid home care							
Never	11 962 (100)	0	<.001	NA	NA	NA	.66
Round 1	0	850 (41.4)		399 (41.5)	45 (36.6)	406 (42.0)	
Round 2	0	387 (18.9)		174 (18.1)	31 (25.2)	182 (18.8)	
Round 3	0	408 (19.9)		190 (19.8)	23 (18.7)	195 (20.2)	
Round 4	0	262 (12.8)		126 (13.1)	18 (14.6)	118 (12.2)	
Round 5	0	144 (7.0)		73 (7.6)	6 (4.9)	65 (6.7)	

Abbreviation: GED, general education diploma; IQR, interquartile range; NA, not applicable.

^a^ The “other” race and ethnicity category included American Indian or Native American, Alaska Native, and Native Hawaiian or Pacific Islander individuals. In 1996 to 2001 and 2013 to 2017, Native Hawaiian or Pacific Islander individuals were included in the Asian category.

Results of the primary difference-in-difference analyses are shown in Table 2 and Table 3. Among likely caregivers, onset of Medicaid home care in the household was associated with an improvement in self-rated mental health of 0.08 standard deviations (95% CI, 0.01-0.14; *P* = .02) in the fully adjusted model. This was equivalent to a 3.39% improvement (95% CI, 0.05%-6.33%) over their average preonset self-rated mental health. Equivalent results on the original (unstandardized) scale are in the eResults and eTable 4 in the Supplement. There was no association between onset of Medicaid home care and self-rated physical health (<0.001 standard deviations; 95% CI, −0.06 to 0.06; *P* = .99).

**Table 2.** Difference-in-Difference Models: Parameter Estimates for Self-rated Mental Health^a^

Variable	Unadjusted			Adjusted for individual-level covariates			Adjusted for individual- and household-level covariates		
	Coef	SE (95% CI)	*P* value	Coef	SE (95% CI)	*P* value	Coef	SE (95% CI)	*P* value
**Medicaid home care onset**									
Likely caregiver^b^	0.06	0.03 (−0.005 to 0.13)	.07	0.06	0.03 (−0.01 to 0.12)	.08	0.08	0.03 (0.01 to 0.14)	.02
Disabled nonrecipient	−0.12	0.12 (−0.35 to 0.12)	.34	−0.11	0.12 (−0.34 to 0.13)	.38	−0.10	0.12 (−0.33 to 0.13)	.39
Medicaid home care recipient	−0.06	0.04 (−0.14 to 0.01)	.12	−0.03	0.04 (−0.11 to 0.04)	.38	−0.03	0.04 (−0.11 to 0.04)	.36
**Round (reference, 1)**									
Round 2	−0.10	0.01 (−0.11 to −0.08)	<.001	−0.09	0.01 (−0.11 to −0.08)	<.001	−0.09	0.01 (−0.11 to −0.06)	<.001
Round 3	−0.14	0.01 (−0.16 to −0.12)	<.001	−0.13	0.01 (−0.15 to −0.12)	<.001	−0.13	0.01 (−0.15 to −0.12)	<.001
Round 4	−0.19	0.01 (−0.20 to −0.17)	<.001	−0.18	0.01 (−0.20 to −0.16)	<.001	−0.18	0.01 (−0.20 to −0.17)	<.001
Round 5	−0.20	0.01 (−0.22 to −0.18)	<.001	−0.20	0.01 (−0.21 to −0.18)	<.001	−0.20	0.01 (−0.22 to −0.18)	<.001
**Emergency department visits (reference, none)**									
1	NC	NC	NC	−0.01	0.01 (−0.04 to 0.01)	.27	−0.01	0.01 (−0.04 to 0.01)	.30
2	NC	NC	NC	−0.06	0.02 (−0.11 to −0.01)	.02	−0.058	0.02 (−0.11 to −0.01)	.02
3	NC	NC	NC	0.01	0.05 (−0.09 to 0.11)	.84	0.02	0.05 (−0.08 to 0.12)	.67
4	NC	NC	NC	−0.06	0.08 (−0.21 to 0.09)	.43	−0.06	0.08 (−0.21 to 0.09)	.46
≥5	NC	NC	NC	−0.04	0.08 (−0.19 to 0.11)	.58	−0.04	0.08 (−0.19 to 0.12)	.65
**Hospitalizations (reference, none)**									
1	NC	NC	NC	−0.06	0.01 (−0.09 to −0.03)	<.001	−0.06	0.01 (−0.09 to −0.03)	<.001
2	NC	NC	NC	−0.07	0.03 (−0.13 to −0.01)	.02	−0.07	0.03 (−0.13 to −0.01)	.02
3	NC	NC	NC	−0.19	0.06 (−0.31 to −0.08)	.001	−0.21	0.06 (−0.32 to −0.09)	<.001
4	NC	NC	NC	−0.24	0.08 (−0.39 to −0.08)	.002	−0.25	0.08 (−0.39 to −0.10)	.001
≥5	NC	NC	NC	−0.20	0.13 (−0.46 to 0.07)	.14	−0.21	0.14 (−0.48 to 0.05)	.12
No. of nights in hospital	NC	NC	NC	−0.002	0.001 (−0.003 to −0.001)	.006	−0.002	0.001 (−0.003 to −0.001)	.006
Unemployed at interview date	NC	NC	NC	−0.11	0.02 (−0.14 to −0.08)	<.001	−0.09	0.02 (−0.13 to −0.06)	<.001
**Family members with fair/poor physical health (reference, none)**									
1	NC	NC	NC	NC	NC	NC	−0.06	0.009 (−0.08 to −0.04)	<.001
2	NC	NC	NC	NC	NC	NC	−0.15	0.02 (−0.18 to −0.11)	<.001
3	NC	NC	NC	NC	NC	NC	−0.241	0.04 (−0.31 to −0.17)	<.001
4	NC	NC	NC	NC	NC	NC	−0.22	0.09 (−0.39 to −0.04)	.02
≥5	NC	NC	NC	NC	NC	NC	−0.34	0.14 (−0.61 to −0.06)	.02
**Family members with fair/poor mental health (reference, none)**									
1	NC	NC	NC	NC	NC	NC	−0.20	0.01 (−0.22 to −0.18)	<.001
2	NC	NC	NC	NC	NC	NC	−0.38	0.02 (−0.42 to −0.33)	<.001
3	NC	NC	NC	NC	NC	NC	−0.45	0.05 (−0.55 to −0.36)	<.001
4	NC	NC	NC	NC	NC	NC	−0.59	0.13 (−0.85 to −0.32)	<.001
≥5	NC	NC	NC	NC	NC	NC	−0.70	0.24 (−1.17 to −0.23)	.003
≥1 Family members went to emergency department (vs none)	NC	NC	NC	NC	NC	NC	−0.008	0.009 (−0.03 to 0.01)	.38
≥1 Family members hospitalized (vs none)	NC	NC	NC	NC	NC	NC	0.005	0.01 (−0.01 to 0.02)	.65
Someone in household employed (vs no one)	NC	NC	NC	NC	NC	NC	0.03	0.02 (−0.01 to 0.06)	.14
Constant	0.004	0.005 (−0.006 to 0.015)	.41	0.09	0.01 (0.06 to 0.11)	<.001	0.16	0.02 (0.12 to 0.21)	<.001

Abbreviations: Coef, coefficient; NC, not calculated.

^a^ Analytic population includes all adults living in households with at least 1 disabled adult, 1996 to 2017.

^b^ Medicaid home care onset: “likely caregiver” is the primary difference-in-difference estimate of interest: the association between onset of Medicaid home care in the household and self-rated mental health among likely caregivers.

**Table 3.** Difference-in-Difference Models: Parameter Estimates for Self-rated Physical Health^a^

Variable	Unadjusted			Adjusted for individual-level covariates			Adjusted for individual- and household-level covariates		
	Coef	SE (95% CI)	*P* value	Coef	SE (95% CI)	*P* value	Coef	SE (95% CI)	*P* value
Medicaid home care onset									
Likely caregiver^b^	0.002	0.03 (−0.06 to 0.06)	.96	−0.003	0.03 (−0.06 to 0.06)	.93	0	0.03 (−0.06 to 0.06)	.99
Disabled nonrecipient	0.05	0.08 (−0.11 to 0.22)	.52	0.08	0.08 (−0.08 to 0.24)	.34	0.08	0.08 (−0.07 to 0.24)	.30
Medicaid home care recipient	−0.10	0.03 (−0.16 to −0.03)	.002	−0.04	0.03 (−0.10 to 0.02)	.22	−0.04	0.03 (−0.10 to 0.02)	.25
**Round (reference, 1)**									
Round 2	−0.01	0.01 (−0.03 to 0.001)	.07	0	0.01 (−0.01 to 0.01)	.95	−0.005	0.01 (−0.02 to 0.01)	.46
Round 3	−0.03	0.01 (−0.04 to −0.01)	<.001	−0.01	0.01 (−0.03 to 0.002)	.08	−0.02	0.01 (−0.04 to −0.01)	.003
Round 4	−0.06	0.01 (−0.08 to −0.04)	<.001	−0.04	0.01 (−0.06 to −0.03)	<.001	−0.05	0.01 (−0.07 to −0.04)	<.001
Round 5	−0.06	0.01 (−0.08 to −0.05)	<.001	−0.05	0.01 (−0.07 to −0.03)	<.001	−0.06	0.01 (−0.08 to −0.05)	<.001
**Emergency department visits (reference, none)**									
1	NC	NC	NC	−0.09	0.01 (−0.12 to −0.07)	<.001	−0.09	0.01 (−0.12 to −0.07)	<.001
2	NC	NC	NC	−0.18	0.02 (−0.22 to −0.13)	<.001	−0.17	0.02 (−0.22 to −0.13)	<.001
3	NC	NC	NC	−0.17	0.04 (−0.26 to −0.09)	<.001	−0.17	0.04 (−0.25 to −0.08)	<.001
4	NC	NC	NC	−0.23	0.06 (−0.35 to −0.11)	<.001	−0.23	0.06 (−0.35 to −0.11)	<.001
≥5	NC	NC	NC	−0.20	0.07 (−0.34 to −0.06)	.004	−0.19	0.07 (−0.33 to −0.06)	.006
**Hospitalizations (reference, none)**									
1	NC	NC	NC	−0.15	0.01 (−0.17 to −0.12)	<.001	−0.15	0.01 (−0.17 to −0.12)	<.001
2	NC	NC	NC	−0.17	0.03 (−0.22 to −0.12)	<.001	−0.17	0.03 (−0.22 to −0.12)	<.001
3	NC	NC	NC	−0.22	0.05 (−0.31 to −0.13)	<.001	−0.24	0.05 (−0.33 to −0.14)	<.001
4	NC	NC	NC	−0.33	0.06 (−0.46 to −0.21)	<.001	−0.33	0.06 (−0.46 to −0.21)	<.001
≥5	NC	NC	NC	−0.40	0.12 (−0.64 to −0.17)	.001	−0.41	0.12 (−0.64 to −0.17)	.001
No. of nights in hospital	NC	NC	NC	−0.003	0.001 (−0.004 to −0.002)	<.001	−0.003	0.001 (−0.004 to −0.002)	<.001
Unemployed at interview date	NC	NC	NC	−0.14	0.02 (−0.17 to −0.11)	<.001	−0.13	0.02 (−0.17 to −0.10)	<.001
**Family members with fair/poor physical health (reference, none)**									
1	NC	NC	NC	NC	NC	NC	−0.15	0.008 (−0.16 to −0.13)	<.001
2	NC	NC	NC	NC	NC	NC	−0.26	0.02 (−0.29 to −0.23)	<.001
3	NC	NC	NC	NC	NC	NC	−0.31	0.04 (−0.39 to −0.24)	<.001
4	NC	NC	NC	NC	NC	NC	−0.33	0.08 (−0.49 to −0.18)	<.001
≥5	NC	NC	NC	NC	NC	NC	−0.781	0.11 (−1.00 to −0.56)	<.001
**Family members with fair/poor mental health (reference, none)**									
1	NC	NC	NC	NC	NC	NC	−0.06	0.008 (−0.08 to −0.04)	<.001
2	NC	NC	NC	NC	NC	NC	−0.16	0.02 (−0.20 to −0.12)	<.001
3	NC	NC	NC	NC	NC	NC	−0.17	0.05 (−0.27 to −0.08)	<.001
4	NC	NC	NC	NC	NC	NC	−0.32	0.13 (−0.58 to −0.07)	.01
≥5	NC	NC	NC	NC	NC	NC	−0.12	0.19 (−0.49 to 0.25)	.52
≥1 Family members went to emergency department (vs none)	NC	NC	NC	NC	NC	NC	0.001	0.008 (−0.01 to 0.02)	.87
≥1 Family members hospitalized (vs none)	NC	NC	NC	NC	NC	NC	0.03	0.01 (0.01 to 0.05)	<.001
Someone in household employed (vs no one)	NC	NC	NC	NC	NC	NC	0.01	0.02 (−0.03 to 0.04)	.68
Constant	−0.15	0.005 (−0.16 to −0.14)	<.001	−0.04	0.01 (−0.06 to −0.01)	.002	0.06	0.02 (0.02 to 0.10)	.005

Abbreviations: Coef, coefficient; NC, not calculated.

^a^ Analytic population includes all adults living in households with at least one disabled adult, 1996 to 2017.

^b^ Medicaid home care onset: “likely caregiver” is the primary difference-in-difference estimate of interest: the association between onset of Medicaid home care in the household and self-rated mental health among likely caregivers.

**Figure 1.**
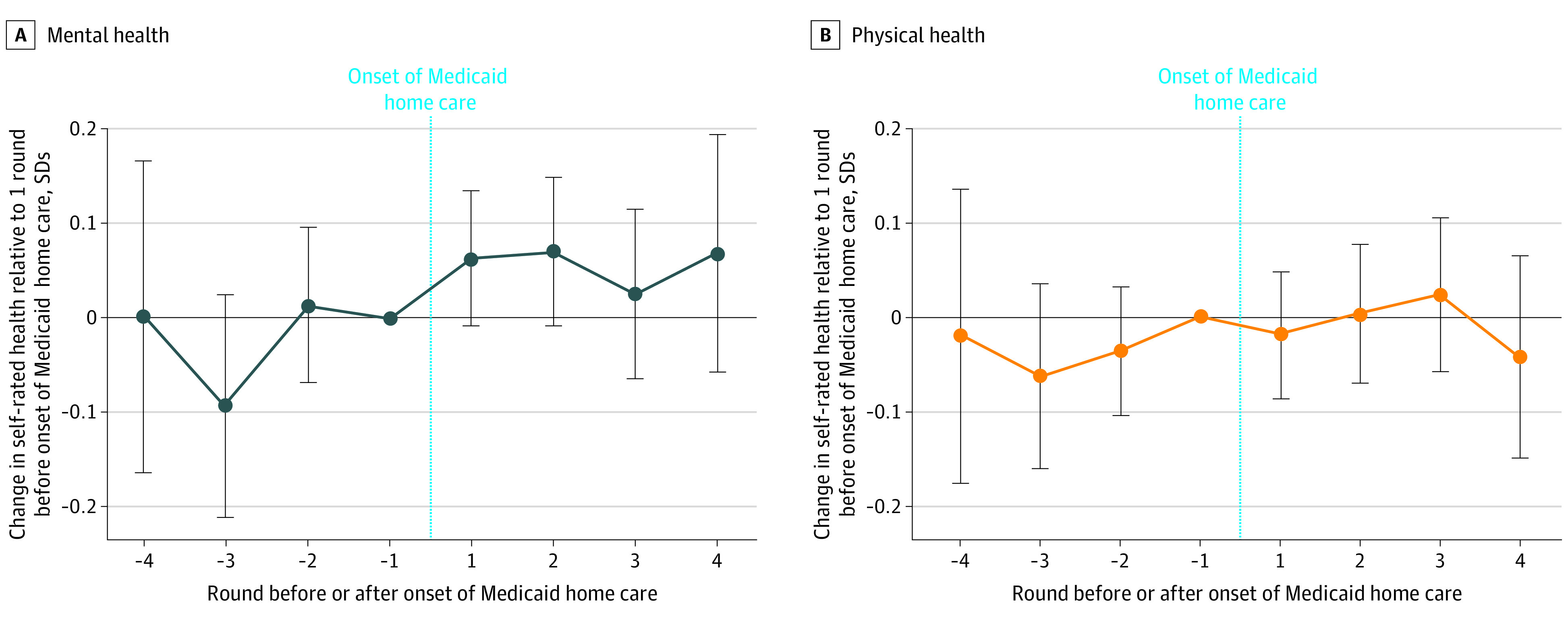
Self-rated Health Among Likely Caregivers Over Time, Relative to Onset of Medicaid Home Care Results shown for all likely caregivers interviewed from 1996 to 2017. Change in self-rated health over time is in standard deviations (SDs) and is relative to the round before onset of Medicaid home care, above and beyond time trends in health observed in the full population. Negative numbers on the x-axis indicate rounds prior to onset of Medicaid home care; positive numbers indicate rounds after onset. Rounds are approximately 6 months apart. The error bars indicate 95% confidence intervals.

Results of the event study model are in Figure 1; eTables 5 and 6, and eFigure 3 in the Supplement. This model showed no trend in likely caregivers’ self-rated mental health (F statistic = 1.10; *P* = .35) or self-rated physical health (F statistic = 0.65; *P* = .58) prior to the onset of Medicaid home care, indicating the parallel pretrends assumption was upheld. Qualitatively, there was sustained self-rated mental health improvement among likely caregivers after the onset of Medicaid home care, with no apparent change in self-rated physical health.

**Figure 2.**
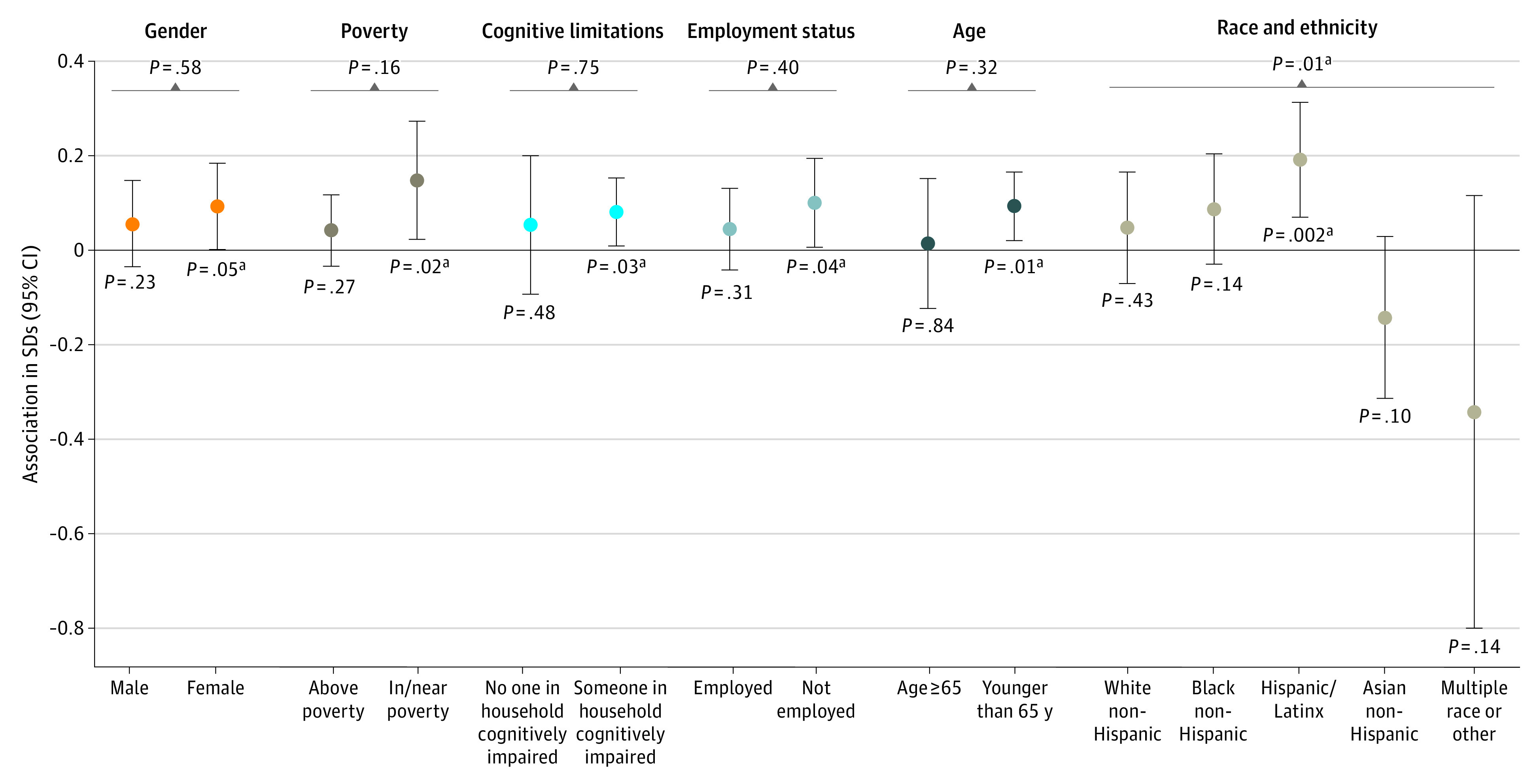
Association Between Onset of Medicaid Home Care and Self-rated Mental Health, by Demographic Group Results shown for all likely caregivers interviewed from 1996 to 2017. All estimates are in standard deviations (SDs) and are from fully adjusted models. The error bars indicate 95% confidence intervals. ^a^Indicates significant results.

Results of the exploratory stratified difference-in-difference analyses are in Figure 2 and eTable 7 in the Supplement. The analysis was underpowered to assess whether there were statistically significant differences in associations by demographic category, but we observed notable trends suggesting association measure modification. Among likely caregivers who were women, Medicaid home care was associated with statistically significant self-rated mental health improvement (0.09 standard deviations; 95% CI, 0.001-0.18; *P* = .05), an average improvement over baseline of 4.2% (95% CI, 0.04%-8.24%); among men, the association was smaller and not statistically significant (0.06 standard deviations; 95% CI, −0.03 to 0.15; *P* = .23). Similarly, the association was larger and statistically significant among caregivers for people with cognitive impairments (0.08 standard deviations; 95% CI, 0.01-0.15; *P* = .03), caregivers living in or near poverty (0.15 standard deviations; 95% CI, 0.02-0.27; *P* = .02), unemployed caregivers (0.10 standard deviations; 95% CI, 0.005-0.19; *P* = .04), and caregivers younger than 65 years (0.09 standard deviations; 95% CI, 0.02-0.17; *P* = .01). The association was smaller and not statistically significant among caregivers for people without cognitive impairments, caregivers living above poverty, employed caregivers, and caregivers older than 65 years. There was statistically significant association measure modification by race or ethnicity. Among Hispanic/Latinx caregivers, onset of Medicaid home care was associated with a 0.19 standard deviation improvement in self-rated mental health (95% CI, 0.07-0.31; *P* = .002). Among Black non-Hispanic caregivers, this association was only 0.09 standard deviations and not significant (95% CI, −0.03 to 0.204; *P* = .14); among White non-Hispanic caregivers, the association was even smaller (0.05 standard deviations; 95% CI, −0.07 to 0.16; *P* = .43).

Sensitivity analysis results are in eResults, eTables 8-11, and eFigure 4 in the Supplement.

## Discussion

In this cohort study, we found that Medicaid home care not only served its direct recipients, but also indirectly benefited their caregivers. Caregivers’ self-rated mental health improved after the onset of home care, gains that were observed 1 to 6 months after onset and appeared to be sustained over time. The initial improvement was approximately equivalent to an 8% decrease in the odds of screening positive for depression on the PHQ-2 or a 9% decrease in the odds of screening positive for severe psychological distress on the Kessler-6 scale (eResults in the Supplement). It was similar in magnitude to the decrease in mental health associated with unemployment or recent hospitalization (though our models were not designed to estimate causal effects for these covariates). Moreover, the association with improved mental health was nearly twice as large among certain socially disadvantaged populations. These results were consistent with prior research showing that women provide more and higher-intensity care, that Black and Latinx people are more likely to bear multiple caregiving responsibilities in paid and unpaid roles, that caregiver burden is greater for socioeconomically disadvantaged people and caregivers for people with cognitive impairments, and that younger caregivers are often members of the “sandwich generation,” with simultaneous obligations to provide childcare and eldercare.^8,19,71,78,79^

We found no spillover association for self-rated physical health. Prior research shows that while mental health^88,89,90^ and some biomarkers^21^ respond quickly to changes in caregiver burden or circumstances, many physical health changes occur over years, not months.^22,23,24^ Since our study only assessed short-term changes, subsequent research should examine whether Medicaid home care affects longer-term physical health.

This study had several strengths, particularly the use of longitudinal data and person-level difference-in-difference models. Although no observational study can provide definitive causal evidence, person-level difference-in-difference models are a rigorous method of reducing confounding. This method also eliminates selection bias due to missing data or loss to follow-up; although such issues might reduce estimates’ generalizability, they will not bias within-person estimates. Because outcomes were always measured after exposures, reverse causation was unlikely. Thus, the primary threat to internal validity is time-varying confounding, which we addressed by applying detailed health care utilization and socioeconomic information to control for likely confounders. Because confounding by socioeconomic status and prior health would create a negative association between Medicaid home care and mental health, it is exceedingly unlikely that the positive association we observe is due to confounding.

### Limitations

Although our study had strong internal validity, it was not generalizable to all caregivers. People who receive Medicaid home care have lower socioeconomic status and worse health than the general population; MEPS also oversamples lower-income populations and people of color.^82^ Our secondary analyses suggested that these populations benefited more from Medicaid home care than others, and our sensitivity analyses showed that when we weighted our study population to reflect national demographics, our primary association attenuated. Our results also may not generalize to caregivers who live in other households. Thus, our results should not be interpreted as the national population-average effect of Medicaid home care, but as evidence that Medicaid home care spills over to at least some caregivers, particularly those who bear the highest burden of care.

Because self-identified caregiver status was unavailable, this study examined “likely caregivers,” diluting the effect of Medicaid home care on true caregivers and excluding caregivers who themselves have disabilities. Data on all household members were reported by a single household respondent, not by each individual, potentially inducing further measurement error and bias toward the null. We defined Medicaid home care as a single, binary exposure; we did not investigate the role of heterogeneity in the volume, type, and duration of services, nor did we investigate long-term associations. Future longitudinal studies should identify caregivers; interview caregiver-care recipient dyads, including non-coresident dyads; explore different types and amounts of Medicaid home care; and use intersectional approaches in larger sample sizes to assess whether particular overlapping racial or ethnic, gender, and socioeconomic groups may benefit most from publicly funded home care.

## Conclusions

In this cohort study, family caregivers’ self-reported mental health improved after the onset of Medicaid home care. As the US population ages and the number of available family caregivers shrinks, policy makers must address a growing caregiving crisis.^1^ Our results suggest publicly funded home care is part of the solution. Although past research has explored Medicaid home care’s benefits for its direct recipients, to our knowledge, ours is the first to show that these programs are associated with better mental health for family caregivers—particularly those who are most disadvantaged. When assessing policies like President Biden’s proposed expansion of Medicaid home- and community-based services,^91^ policy makers should consider not only health benefits for direct recipients, but also spillover health benefits for caregivers.

## Notes

Supplement. eMethods eResults eTable 1. Comparison of demographic and health characteristics of study participants with and without missing data eTable 2. Supplemental baseline demographic and health characteristics of adults in households with at least one disabled adult, 1996-2017 eTable 3. Comparison of never disabled adults (likely caregivers) living in households already receiving Medicaid home care at baseline to those with new-onset Medicaid home care eTable 4. Difference-in-difference models: parameter estimates for self-rated mental health, original scale (unstandardized) eTable 5. Event study models: parameter estimates for self-rated mental health over time eTable 6. Event study models: parameter estimates for self-rated physical health over time eTable 7. Association between Medicaid home care onset and caregivers’ self-rated mental health, by demographic variables eTable 8. Weighted difference-in-difference models: parameter estimates for self-rated mental health eTable 9. Weighted difference-in-difference models: parameter estimates for self-rated physical health eTable 10. Comparison of demographic and health characteristics of study participants with and without high survey weights eTable 11. Association between self-rated mental health and clinically interpretable measures of mental health disorders eFigure 1. Exposure status over time among Medicaid home care recipients eFigure 2. Exposure status over time among all individuals in households ever exposed to Medicaid home care eFigure 3. Self-rated health over time relative to onset of Medicaid home care, by household role eFigure 4. Histogram of the longitudinal weights eReferences
