# Supplementary material for: Association Between New-Onset Medicaid Home Care and Family Caregivers’ Health
Source: JAMA Health Forum. 2021 Sep 17;2(9):e212671. doi: 10.1001/jamahealthforum.2021.2671 (PMC8796991; doi:10.1001/jamahealthforum.2021.2671)
Supplement: Supplement. — eMethods eResults eTable 1. Comparison of demographic and health characteristics of study participants with and without missing data eTable 2. Supplemental baseline demographic and health characteristics of adults in households with at least one disabled adult, 1996-2017 eTable 3. Comparison of never disabled adults (likely caregivers) living in households already receiving Medicaid home care at baseline to those with new-onset Medicaid home care eTable 4. Difference-in-difference models: parameter estimates for self-rated mental health, original scale (unstandardized) eTable 5. Event study models: parameter estimates for self-rated mental health over time eTable 6. Event study models: parameter estimates for self-rated physical health over time eTable 7. Association between Medicaid home care onset and caregivers’ self-rated mental health, by demographic variables eTable 8. Weighted difference-in-difference models: parameter estimates for self-rated mental health eTable 9. Weighted difference-in-difference models: parameter estimates for self-rated physical health eTable 10. Comparison of demographic and health characteristics of study participants with and without high survey weights eTable 11. Association between self-rated mental health and clinically interpretable measures of mental health disorders eFigure 1. Exposure status over time among Medicaid home care recipients eFigure 2. Exposure status over time among all individuals in households ever exposed to Medicaid home care eFigure 3. Self-rated health over time relative to onset of Medicaid home care, by household role eFigure 4. Histogram of the longitudinal weights eReferences [file jamahealthforum-e212671-s001.pdf]

Supplemental Online Content

Unger ES, Grabowski DC, Chen JT, Berkman LF. Association between new-onset Medicaid home care and family caregivers' health. *JAMA Health Forum*. 2021;2(9):e212671. doi:10.1001/jamahealthforum.2021.2671

eMethods
eResults
eTable 1. Comparison of demographic and health characteristics of study participants with and without missing data
eTable 2. Supplemental baseline demographic and health characteristics of adults in households with at least one disabled adult, 1996-2017
eTable 3. Comparison of never disabled adults (likely caregivers) living in households already receiving Medicaid home care at baseline to those with new-onset Medicaid home care
eTable 4. Difference-in-difference models: parameter estimates for self-rated mental health, original scale (unstandardized)
eTable 5. Event study models: parameter estimates for self-rated mental health over time
eTable 6. Event study models: parameter estimates for self-rated physical health over time
eTable 7. Association between Medicaid home care onset and caregivers' self-rated mental health, by demographic variables
eTable 8. Weighted difference-in-difference models: parameter estimates for self-rated mental health
eTable 9. Weighted difference-in-difference models: parameter estimates for self-rated physical health
eTable 10. Comparison of demographic and health characteristics of study participants with and without high survey weights
eTable 11. Association between self-rated mental health and clinically interpretable measures of mental health disorders
eFigure 1. Exposure status over time among Medicaid home care recipients
eFigure 2. Exposure status over time among all individuals in households ever exposed to Medicaid home care
eFigure 3. Self-rated health over time relative to onset of Medicaid home care, by household role
eFigure 4. Histogram of the longitudinal weights
eReferences

This supplemental material has been provided by the authors to give readers additional information about their work.

© 2021 Unger ES et al. *JAMA Health Forum.*

eMethods

Population

Dataset details
Each household in the Medical Expenditures Panel Survey (MEPS) was interviewed approximately every six months over the course of two years, for a total of five waves of data collection. While each panel of was surveyed over the course of a two-year period, a new panel began every year, such that the interview periods for subsequent panels overlapped. For example, households in panel 20 of MEPS were surveyed five times over the years 2015-2016, while households in panel 21 were surveyed five times over the years 2016-2017.

Exclusion criteria details
We began with the 331,202 individuals included in the MEPS longitudinal data files. These files exclude all individuals lost to follow up (about 10% of the population; exact share varies by panel) but include individuals who enter or leave the survey because they are born, die, are institutionalized, join a surveyed household, leave the country, or otherwise leave the scope of the survey. To define stable households, we excluded the 4.2% of the population who lived two or more different households over the course of the survey.

We then limited our analytic sample to the 21,184 individuals who had limitations in their activities of daily living (ADLs) in at least one wave or who lived in the same household (referred to in MEPS as a "reporting unit") as someone with ADL limitations in at least one wave. ADL limitations were assessed with a single global question, in which a household respondent was asked "Does anyone in this household receive help or supervision with personal care such as bathing, dressing, or getting around the house because of an impairment or a physical or mental health problem?" If the respondent said yes, they were then asked to indicate which household members needed such assistance.

We excluded people who lived in one-person households, because these households had no potential for within-household caregiving. We excluded children under age 21, because our study focused on caregiving for adults and because children are less likely to act as caregivers. Finally, we excluded individuals who were missing data for any covariates or outcome measures. People were excluded from the model in any round in which they had missing data, but were only excluded completely from the model if they had missing data in every round.

© 2021 Unger ES et al. *JAMA Health Forum.*

Variables

Exposure definition
MEPS defines home health services as health-related services received in the home from any provider, including home health agencies, independent/self-employed providers, and "informal" (unpaid) providers who reside outside of the household. For paid home health services, MEPS categorizes payors as:

1. Out-of-pocket by User or Family,
2. Medicare,
3. Medicaid,
4. Private Insurance,
5. Veterans Administration/CHAMPVA, excluding TRICARE,
6. TRICARE,
7. Other Federal Sources - includes Indian Health Service, military treatment facilities, and other care by the federal government,
8. Other State and Local Sources - includes community and neighborhood clinics, state and local health departments, and state programs other than Medicaid,
9. Workers' Compensation, and
10. Other Unclassified Sources - includes sources such as automobile, homeowner's, and liability insurance, and other miscellaneous or unknown sources.
11. Other Private - any type of private insurance payments reported for persons not reported to have any private health insurance coverage during the year as defined in MEPS, and
12. Other Public - Medicare/Medicaid payments reported for persons who were not reported to be enrolled in the Medicare/Medicaid program at any time during the year.[1]

The MEPS documentation further notes that "Though relatively small in magnitude, data users/analysts should exercise caution when interpreting the expenditures associated with these two additional sources of payment ["other private" and "other public"]. While these payments stem from apparent inconsistent responses to health insurance and source of payment questions in the survey, some of these inconsistencies may have logical explanations. […] Some of the "other public" payments may stem from confusion between Medicaid and other state and local programs or may be from persons who were not enrolled in Medicaid, but were presumed eligible by a provider who ultimately received payments from the public payer." [1]

Because it is likely that services categorized as "other public" were in fact paid for by Medicaid, we defined Medicaid home care services as including any services coded as paid for by Medicaid or "other public" payors. Note that this definition does not include major non-Medicaid public payors, including Medicare, VA, TRICARE, and state and local programs.

Covariate selection
All the time-varying confounders included in our model were available in every wave. Other socioeconomic variables (e.g. income) were available only on an annual basis, so were not included in the model as time-varying confounders. Questions on ADL and IADL limitations were not asked in every wave, so these variables were also not included as time-varying confounders.

All time-varying confounders included were from the same wave in which the outcome was measured. Because our models were conditional on the individual and individuals each had a small number of data points (a maximum of five), we found that adding lagged time-varying covariates (that is, including these measures from the round prior) created a substantial regression to the mean problem which caused the coefficients on these covariates to be in the opposite direction of the known relationship between that covariate and the outcome. Thus, we did not include any lagged time-varying covariates in the model.

© 2021 Unger ES et al. *JAMA Health Forum.*

Model

Estimating Equations

We used person-level difference-in-difference models to assess within-person change in the outcomes associated with within-person onset of the exposure. Our primary model was as follows:

$$Y_{it} = \beta_0 + \alpha_t + \gamma_i + \beta_1 post_t * homecare_i * HH\_role_i + \beta_2 L_{it} + \epsilon_{it}$$

i indexes individuals and *t* indexes time in rounds. α_t is a vector of round indicator variables (fixed effects), γ_i is a vector of person indicator variables (fixed effects), L_{it} is a vector of time-varying confounders, and Y_{it} is the outcome. $post_t * homecare_i$ is the difference-in-difference estimator: it is zero in rounds in which no one in the household receives Medicaid home care and one in the first round in which someone in the household receives Medicaid home care. HH_role_i indicates whether an individual is a non-disabled adult (likely caregiver), disabled adult, or Medicaid home care recipient.

Individuals who were never exposed to Medicaid home care had a value of zero for $post_t * homecare_i$ in all waves. Thus, they did not contribute to the difference-in-difference effect estimate; they did, however, contribute to the estimates of overall time trends and other covariates, and, as such, acted as a comparator group for the difference-in-difference estimate. Individuals who already received Medicaid home care in wave 1 also did not contribute to the effect estimate because they had no pre-exposure outcome and dropped out of the model after the first wave, so their change in outcome could not be assessed.

Our event study model was as follows:

$$Y_{it} = \beta_0 + \alpha_t + \gamma_i + \sum_{p=-4}^{-2} \alpha_p\left(mhc\_period_{ip}\right) * HH\_role_i + \sum_{p=1}^{5} \alpha_p\left(mhc\_period_{ip}\right) * HH\_role_i + \beta_2 L_{it} + \epsilon_{it}$$

Where subscript p indexes the event period (number of rounds before or after onset of Medicaid home care), and mhc_period_{ip} denotes a series of binary indicators equal to 1 if an observation occurred in time period p for person *i*. For individuals who lived in households that never received Medicaid home care, mhc_period_{ip} was equal to zero for all p. For individuals who lived in households that already received Medicaid home care in round one, it was impossible to determine the event period, because it was unknown how long these households had received Medicaid home care before the survey began. Thus, these individuals also received a value of zero for mhc_period_{ip} for all p and, like in the main difference-in-difference model, did not contribute to the effect estimates. All other notation is as above.

Our model to assess effect measure modification was as follows:

$$Y_{it} = \beta_0 + \alpha_t + \gamma_i + \beta_1 post_t * homecare_i * HH_{role_i} + \beta_2 post_t * homecare_i * HH_{role_i} * modifier_i + \beta_2 L_{it} + \epsilon_{it}$$

Where $modifier_i$ indicates the effect measure modifier of interest (e.g. unemployment.). All other notation is as above.

All results should be interpreted as the within-person change in the outcome associated with the onset of Medicaid home care, above and beyond any changes in the outcome that occurred over time among individuals who did not start Medicaid home care, conditional on all the covariates in the model. In other words, these results reflect the difference between an individual's self-rated health one to six months after the start of Medicaid home care services and their self-rated health in the six-month to two-year period before anyone in their household received Medicaid home care, relative to the difference over this same period for a similar individual whose household members did not begin receiving services in that wave.

Variance estimation

In fixed effects models, it is generally appropriate to use cluster-robust standard errors clustered at the level of the fixed effect – here, at the individual level.[2] However, because the MEPS data derive from a clustered survey design,

© 2021 Unger ES et al. *JAMA Health Forum.*

we also considered clustering the standard errors at the survey cluster level. In order to assess which approach was most appropriate, we tested our primary model three ways: with standard errors clustered at the individual level, with standard errors clustered at the survey primary sampling unit (PSU) level, and with standard errors clustered at the stratum level.

We found that standard errors were largest when we clustered them at the individual level. When we clustered standard errors at the PSU and stratum level, the standard error on the main association decreased by 33% and 3%, respectively. Thus, we proceeded with the most conservative option: standard errors clustered at the individual level.

© 2021 Unger ES et al. *JAMA Health Forum.*

Sensitivity Analyses

Weighted analyses

MEPS provides cross-sectional and longitudinal survey weights to weight the sample population to match national demographics. The cross-sectional survey weights adjust for the oversampling of racial/ethnic minorities and low-income populations in MEPS. The longitudinal weights also adjust for loss-to-follow-up; however, they have a 97% correlation with the cross-sectional weights, suggesting that they primarily adjust for oversampling.

Our primary analysis did not apply the survey weights, as we had no basis for estimating a national average effect of Medicaid home care. We performed a secondary analysis that was equivalent to our primary analysis but with the survey weights applied. We then performed the same analysis but truncated the weights at the 90th percentile, in order to evaluate the impact of using or excluding extremely large weights.

Additionally, we performed descriptive analysis of the survey weights to explore the ways in which weighting vs. not weighting our primary models might affect the results. We described the univariate distribution of the survey weights using summary statistics and a histogram. We assessed how the demographics of individuals with large weights (90th percentile or higher) differed from the rest of the population using chi-squared tests.

Analyses of clinical significance of the outcome

Our primary outcomes of interest were self-rated overall mental and physical health. We used these outcomes rather than more clinically-applicable health outcomes because they were available for all individuals in every wave. Additionally, for our primary analysis, we operationalized these outcomes as continuous, because this allowed us to use conditional likelihood models without dropping individuals whose outcomes remained the same for the entire study period. (Conditional logistic and ordinal regression would drop all individuals who have no variation in their outcome during the study period.)

Although these outcomes worked well for modelling purposes, their clinical interpretation is more challenging. To provide readers with a better sense of the clinical meaning of our primary outcome, we assessed the relationship between self-rated mental health and more commonly used screening tools for mental health disorders in our study population.

Beginning in 2004, we had access to two additional measures of mental health: the Patient Health Questionnaire-2 (PHQ-2) and the Kessler-6. These questions were only asked during round 2 and 4 interviews, so they could not be used in our primary model. However, they provide clinically useful metrics of depression (PHQ-2) and severe psychological distress (Kessler-6). Additionally, these questions were asked using a self-administered questionnaire that each adult in the household filled out for themself, rather than by a single household respondent who answered for everyone in the household. Thus, these measures are less prone to measurement error than the global self-rated mental health measure. Details on the PHQ-2 and Kessler-6, as well as the relationship between these measures and self-rated mental health, are available elsewhere.[3]

Using data from all members of our study population in all rounds in which the PHQ-2 and Kessler-6 data were available, we assessed the relationship between an 0.01 standard deviation change in the self-rated mental health score and the log odds of being depressed or having severe psychological distress using logistic regression with robust standard errors. The estimating equation was as follows:

$$logit(P[Y_{it} = 1]) = \beta_0 + \beta_1 SRMH_{it}$$

where Y_{it} is the outcome for person *i* at time *t* (either screening positive for depression on the PHQ-2 scale or screening positive for severe psychological distress on the Kessler-6 scale) and $SRMH_{it}$ is the standardized self-rated mental health score, in increments of 0.01 of a standard deviation, for person *i* at time *t.*

We operationalized the exposure as an 0.01 standard deviation change in the self-rated mental health score in order to make it straightforward for the reader to translate any association estimate from our main models into an approximate estimate of clinical significance using the results of this model. We did not condition on any covariates, person, or round, as we sought to simply assess the bivariate relationship between these variables. We defined

© 2021 Unger ES et al. *JAMA Health Forum.*

depression as a score of 3 or higher on the PHQ-2 and severe psychological distress as a score of 13 or higher on the Kessler-6, typical cut-offs used elsewhere in the literature.[3]

Results of this model should be used only to assess the validity of the self-rated mental health measure and to provide the reader with a general sense of the clinical meaning of a certain magnitude change in self-rated mental health. They should not be used to make claims about the relationship between onset of Medicaid home care and either depression or severe psychological distress. Our models cannot provide a definitive assessment of those relationships, as our models do not account for the uncertainty of the relationship between self-rated mental health and depression or severe psychological distress.

© 2021 Unger ES et al. *JAMA Health Forum.*

eResults

eTable 1 shows a comparison of the demographics of the 50 adults excluded due to missing data versus the analytic population. It should be noted that people living in households that received Medicaid home care were more likely to be excluded due to missingness because these individuals dropped out of the model after the first round of exposure to Medicaid home care; thus, they had fewer rounds of data available and were more likely to have no rounds with complete data.

Certain individuals were included in the model but did not contribute to effect estimates because they did not have both a pre-exposure period and a post-exposure period. This includes all individuals living in households that were never exposed to Medicaid home care, who had no "post" period. It also includes all individuals living in households that were already exposed to Medicaid home care in round one, as these individuals had no "pre" period. A comparison of the demographics of never-disabled adults (likely caregivers) who were already exposed to Medicaid home care in round one (and thus did not contribute to effect estimates) and those who were exposed after round one (and did contribute to effect estimates) can be found in eTable 3.

Results of the primary analysis shown for self-rated mental health on the original scale (unstandardized) are found in eTable 4. Note that the original scale ranges from 1 to 5, with lower numbers indicating better self-rated mental health: 1 indicates excellent health and 5 indicates poor health. On this scale, the average self-rated mental health of likely caregivers in rounds prior to the onset of Medicaid home care in the household was 2.35 (95% CI: 2.28, 2.41). In fully-adjusted models, onset of Medicaid home care was associated with a -0.090 change in self-rated mental health among likely caregivers (95% CI: -0.168, -0.012; p=0.024).

Results of the weighted analyses are found in eTables 8 and 9. In the weighted analysis, the association between onset of Medicaid home care and self-rated mental health was attenuated (0.019 standard deviations; 95% CI -0.065, 0.103; p=0.66).

Results of the descriptive analysis of the weights are found in eFigure 4 and eTable 10. The distribution of the weights was extremely skewed: the median weight was 14,087.64 and the inter-quartile range was 15,500.761, but the 90th percentile was 35,436.57 and the maximum weight was 170,898.9. This indicated that a small number of individuals were up-weighted more than ten times the median individual.

Comparison of individuals with the top 10% of weights to the rest of the population (eTable 10) revealed that individuals with the top 10% of weights were disproportionately older, male, White non-Hispanic, highly educated, high-income, employed, and in good health, and were less likely to live with someone with cognitive limitations. Thus, the individuals with extremely high weights typically fell into the demographic categories that our analysis of effect measure modification found benefitted less from Medicaid home care.

Truncating the weights at the 90th percentile to exclude extreme weights resulted in an estimate of association between onset of Medicaid home care and self-rated mental health similar to our unweighted analysis, though the 95% confidence intervals were slightly wider due to the smaller sample size (0.079 standard deviations; 95% CI: -0.002, 0.160, p=0.06).

Results of the analysis of the clinical significance of self-rated health are found in eTable 11. An 0.01 of a standard deviation improvement in self-rated mental health predicts a 1.09% lower odds of having depression (OR: 0.9891, 95% CI: 0.9886, 0.9896) and a 1.25% lower odds of having severe psychological distress (OR: 0.9875, 95% CI: 0.9869, 0.9882). An 0.075 of a standard deviation improvement in self-rated mental health (the main association between onset of Medicaid home care and likely caregivers' self-rated mental health found in our fully-adjusted primary analysis) thus predicts a 7.92% lower odds of having depression (OR: 0.9208, 95% CI: 0.9174, 0.9243) and a 9.02% lower odds of having severe psychological distress (OR: 0.9098, 95% CI: 0.9057, 0.9146).

© 2021 Unger ES et al. *JAMA Health Forum.*

eTables

eTable 1: Comparison of demographic and health characteristics of study participants with and without missing data

All cells show n (percent). Population comprises all adults living in households with at least one disabled adult interviewed in 1996-2017.

	Excluded due to missing data	In analytic sample	p-value
	N=50	N=14,013	
**Someone in household ever receives Medicaid home care**	21 (42.0%)	2,051 (14.6%)	<0.001
**Household role**			
Never disabled adult	8 (42.1%)	7,232 (51.6%)	
Ever disabled adult	1 (5.3%)	5,815 (41.5%)	
Medicaid home care recipient	10 (52.6%)	966 (6.9%)	<0.001
**Age at baseline**			
Under age 21	0 (0.0%)	252 (1.8%)	
Age 21-34	5 (10.0%)	1,711 (12.2%)	
Age 35-49	8 (16.0%)	2,944 (21.0%)	
Age 50-64	6 (12.0%)	3,870 (27.6%)	
Age 65-79	14 (28.0%)	3,410 (24.3%)	
Age 80+	17 (34.0%)	1,826 (13.0%)	<0.001
**Gender**			
Male	36 (72.0%)	6,187 (44.2%)	
Female	14 (28.0%)	7,826 (55.8%)	<0.001
**Race/ethnicity**			
White non-Hispanic	25 (50.0%)	6,968 (49.7%)	
Hispanic/Latinx	9 (18.0%)	3,141 (22.4%)	
Black non-Hispanic	14 (28.0%)	2,844 (20.3%)	
Asian non-Hispanic	2 (4.0%)	737 (5.3%)	
Multiple race or other	0 (0.0%)	323 (2.3%)	0.53
**Education level**			
Less than or equal to 8th grade	7 (18.4%)	2,491 (17.9%)	
9-12 grade, no diploma	3 (7.9%)	2,110 (15.2%)	
High school diploma or GED	15 (39.5%)	4,911 (35.4%)	
Some college or associate's degree	6 (15.8%)	2,547 (18.3%)	
Bachelor's degree	5 (13.2%)	1,225 (8.8%)	
Graduate school	2 (5.3%)	601 (4.3%)	0.77
**Household income level at baseline**			
Poor/negative	8 (16.0%)	2,681 (19.1%)	
Near poor	11 (22.0%)	1,070 (7.6%)	
Low income	7 (14.0%)	2,795 (19.9%)	
Middle income	14 (28.0%)	4,290 (30.6%)	
High income	10 (20.0%)	3,177 (22.7%)	0.005
**Employed at baseline**	2 (4.3%)	4,412 (31.5%)	<0.001
**Someone else in the household ever has cognitive limitations**	34 (68.0%)	6,337 (45.2%)	0.001
**Self-rated physical health at baseline**			
Excellent	3 (16.7%)	1,838 (13.1%)	
Very good	4 (22.2%)	2,584 (18.4%)	
Good	4 (22.2%)	3,703 (26.4%)	
Fair	3 (16.7%)	3,315 (23.7%)	
Poor	4 (22.2%)	2,573 (18.4%)	0.92
**Self-rated mental health at baseline**			
Excellent	1 (5.9%)	3,443 (24.6%)	
Very good	3 (17.6%)	3,166 (22.6%)	
Good	7 (41.2%)	4,276 (30.5%)	
Fair	2 (11.8%)	2,081 (14.9%)	
Poor	4 (23.5%)	1,047 (7.5%)	0.06

© 2021 Unger ES et al. *JAMA Health Forum.*

eTable 2: Supplemental baseline demographic and health characteristics of adults in households with at least one disabled adult, 1996-2017

All cells show n (percent), except where noted. HH=household. All values shown are at baseline.

	**Full population (n=14,013)**			**Population in households that receive Medicaid home care (n=2,051)**			
	In household that never receives Medicaid home care (n=11,962)	In household that receives Medicaid home care (n=2,051)	p-value	Likely caregiver (n=962)	Disabled non-recipient (n=123)	Medicaid home care recipient (n=966)	p-value
**Age in years, categorical**			0.008				<0.001
Under age 21	235 (2.0%)	17 (0.8%)		14 (1.5%)	0 (0.0%)	3 (0.3%)	
Age 21-34	1,459 (12.2%)	252 (12.3%)		171 (17.8%)	8 (6.5%)	73 (7.6%)	
Age 35-49	2,521 (21.1%)	423 (20.6%)		251 (26.1%)	14 (11.4%)	158 (16.4%)	
Age 50-64	3,280 (27.4%)	590 (28.8%)		324 (33.7%)	35 (28.5%)	231 (23.9%)	
Age 65-79	2,925 (24.5%)	485 (23.6%)		163 (16.9%)	42 (34.1%)	280 (29.0%)	
Age 80+	1,542 (12.9%)	284 (13.8%)		39 (4.1%)	24 (19.5%)	221 (22.9%)	
**Household income level**			<0.001				<0.001
Poor/negative	2,086 (17.4%)	595 (29.0%)		208 (21.6%)	48 (39.0%)	339 (35.1%)	
Near poor	849 (7.1%)	221 (10.8%)		92 (9.6%)	13 (10.6%)	116 (12.0%)	
Low income	2,327 (19.5%)	468 (22.8%)		231 (24.0%)	29 (23.6%)	208 (21.5%)	
Middle income	3,788 (31.7%)	502 (24.5%)		274 (28.5%)	25 (20.3%)	203 (21.0%)	
High income	2,912 (24.3%)	265 (12.9%)		157 (16.3%)	8 (6.5%)	100 (10.4%)	
**Anyone in household employed**	6,889 (57.6%)	1,013 (49.4%)	<0.001	585 (60.8%)	33 (26.8%)	395 (40.9%)	<0.001
**Number of emergency room visits**			0.54				<0.001
None	10,972 (91.7%)	1,860 (90.7%)		917 (95.3%)	113 (91.9%)	830 (85.9%)	
One	797 (6.7%)	154 (7.5%)		39 (4.1%)	7 (5.7%)	108 (11.2%)	
Two	139 (1.2%)	24 (1.2%)		5 (0.5%)	2 (1.6%)	17 (1.8%)	
Three	31 (0.3%)	9 (0.4%)		1 (0.1%)	0 (0.0%)	8 (0.8%)	
Four	11 (0.1%)	2 (0.1%)		0 (0.0%)	1 (0.8%)	1 (0.1%)	
Five or more	12 (0.1%)	2 (0.1%)		0 (0.0%)	0 (0.0%)	2 (0.2%)	
**Number of hospitalizations**			0.26				<0.001
None	11,166 (93.3%)	1,888 (92.1%)		944 (98.1%)	112 (91.1%)	832 (86.1%)	
One	631 (5.3%)	123 (6.0%)		16 (1.7%)	10 (8.1%)	97 (10.0%)	
Two	120 (1.0%)	29 (1.4%)		2 (0.2%)	1 (0.8%)	26 (2.7%)	
Three	29 (0.2%)	8 (0.4%)		0 (0.0%)	0 (0.0%)	8 (0.8%)	
Four	8 (0.1%)	2 (0.1%)		0 (0.0%)	0 (0.0%)	2 (0.2%)	
Five or more	8 (0.1%)	1 (0.0%)		0 (0.0%)	0 (0.0%)	1 (0.1%)	
**Number of HH members in fair/poor self-rated physical health**			<0.001				<0.001
None	6,084 (50.9%)	818 (39.9%)		257 (26.7%)	37 (30.1%)	524 (54.2%)	
One	4,892 (40.9%)	1,013 (49.4%)		540 (56.1%)	80 (65.0%)	393 (40.7%)	
Two	811 (6.8%)	199 (9.7%)		149 (15.5%)	6 (4.9%)	44 (4.6%)	
Three	150 (1.3%)	14 (0.7%)		11 (1.1%)	0 (0.0%)	3 (0.3%)	
Four	17 (0.1%)	5 (0.2%)		4 (0.4%)	0 (0.0%)	1 (0.1%)	
Five or more	8 (0.1%)	2 (0.1%)		1 (0.1%)	0 (0.0%)	1 (0.1%)	
**Number of HH members in fair/poor self-rated mental health**			<0.001				<0.001
None	8,554 (71.5%)	1,300 (63.4%)		515 (53.5%)	67 (54.5%)	718 (74.3%)	
One	2,940 (24.6%)	671 (32.7%)		389 (40.4%)	52 (42.3%)	230 (23.8%)	
Two	397 (3.3%)	69 (3.4%)		51 (5.3%)	4 (3.3%)	14 (1.4%)	
Three	56 (0.5%)	9 (0.4%)		6 (0.6%)	0 (0.0%)	3 (0.3%)	
Four	14 (0.1%)	2 (0.1%)		1 (0.1%)	0 (0.0%)	1 (0.1%)	
Five or more	1 (0.0%)	0 (0.0%)		0 (0.0%)	0 (0.0%)	0 (0.0%)	
**Any HH members with emergency room visits**	1,534 (12.8%)	286 (13.9%)	0.16	177 (18.4%)	20 (16.3%)	89 (9.2%)	<0.001
**Any HH members hospitalized**	1,123 (9.4%)	223 (10.9%)	0.04	158 (16.4%)	14 (11.4%)	51 (5.3%)	<0.001

© 2021 Unger ES et al. *JAMA Health Forum.*

eTable 3: Comparison of never disabled adults (likely caregivers) living in households already receiving Medicaid home care at baseline to those with new-onset Medicaid home care

All cells show n (percent). Population comprises all never-disabled adults living in households with at least one disabled adult that ever received Medicaid home care and were interviewed in 1996-2017.

	Household already receiving Medicaid home care in round 1	Household began receiving Medicaid home care after round 1	p-value
	N=399	N=563	
**Household role**			
Never disabled adult (likely caregiver)	399 (100.0%)	563 (100.0%)	n/a
**Age at baseline**			
Under age 21	0 (0.0%)	14 (2.5%)	
Age 21-34	76 (19.0%)	95 (16.9%)	
Age 35-49	109 (27.3%)	142 (25.2%)	
Age 50-64	132 (33.1%)	192 (34.1%)	
Age 65-79	71 (17.8%)	92 (16.3%)	
Age 80+	11 (2.8%)	28 (5.0%)	0.02
**Gender**			
Male	201 (50.4%)	282 (50.1%)	
Female	198 (49.6%)	281 (49.9%)	0.93
**Race/ethnicity**			
White non-Hispanic	107 (26.8%)	189 (33.6%)	
Hispanic/Latinx	145 (36.3%)	164 (29.1%)	
Black non-Hispanic	124 (31.1%)	155 (27.5%)	
Asian non-Hispanic	16 (4.0%)	43 (7.6%)	
Multiple race or other	7 (1.8%)	12 (2.1%)	0.009
**Education level**			
Less than or equal to 8th grade	60 (15.2%)	96 (17.2%)	
9-12 grade, no diploma	60 (15.2%)	110 (19.7%)	
HS diploma or GED	136 (34.3%)	185 (33.2%)	
Some college or associate's degree	88 (22.2%)	96 (17.2%)	
Bachelor's degree	38 (9.6%)	50 (9.0%)	
Graduate school	14 (3.5%)	21 (3.8%)	0.26
**Household income level at baseline**			
Poor/negative	79 (19.8%)	129 (22.9%)	
Near poor	39 (9.8%)	53 (9.4%)	
Low income	108 (27.1%)	123 (21.8%)	
Middle income	110 (27.6%)	164 (29.1%)	
High income	63 (15.8%)	94 (16.7%)	0.40
**Employed at baseline**	201 (50.4%)	259 (46.0%)	0.18
**Someone else in household ever has cognitive limitations**	334 (83.7%)	437 (77.6%)	0.02
**Self-rated physical health at baseline**			
Excellent	64 (16.0%)	117 (20.8%)	
Very good	83 (20.8%)	108 (19.2%)	
Good	138 (34.6%)	175 (31.1%)	
Fair	82 (20.6%)	126 (22.4%)	
Poor	32 (8.0%)	37 (6.6%)	0.28
**Self-rated mental health at baseline**			
Excellent	121 (30.3%)	176 (31.3%)	
Very good	97 (24.3%)	137 (24.3%)	
Good	135 (33.8%)	173 (30.7%)	
Fair	36 (9.0%)	63 (11.2%)	
Poor	10 (2.5%)	14 (2.5%)	0.77
**First round someone in household receives Medicaid home care**			
Round 1	399 (100.0%)	0 (0.0%)	
Round 2	0 (0.0%)	174 (30.9%)	
Round 3	0 (0.0%)	190 (33.7%)	
Round 4	0 (0.0%)	126 (22.4%)	
Round 5	0 (0.0%)	73 (13.0%)	<0.001

eTable 4: Difference-in-difference models: parameter estimates for self-rated mental health, original scale (unstandardized)
Analytic population includes all adults living in households with at least one disabled adult, 1996-2017. "Medicaid home care onset: Likely caregiver" is the primary difference-in-difference estimate of interest: the association between onset of Medicaid home care in the household and self-rated mental health among likely caregivers.

	Unadjusted				Adjusted for individual-level covariates				Adjusted for individual- and household-level covariates			
	Coef.	SE	95% CI	p	Coef.	SE	95% CI	p	Coef.	SE	95% CI	p
**Medicaid home care onset: Likely caregiver**	-0.073	0.040	[-0.152,0.006]	0.07	-0.070	0.040	[-0.149,0.009]	0.08	-0.090	0.040	[-0.168,-0.012]	0.02
**Medicaid home care onset: Disabled non-recipient**	0.139	0.146	[-0.147,0.425]	0.34	0.127	0.144	[-0.155,0.410]	0.38	0.120	0.141	[-0.156,0.396]	0.39
**Medicaid home care onset: Medicaid home care recipient**	0.074	0.047	[-0.018,0.167]	0.12	0.042	0.047	[-0.051,0.134]	0.38	0.042	0.046	[-0.049,0.133]	0.36
**Round (ref: 1)**												
Round 2	0.117	0.010	[0.098,0.136]	<0.001	0.111	0.010	[0.092,0.130]	<0.001	0.110	0.010	[0.092,0.129]	<0.001
Round 3	0.168	0.010	[0.148,0.187]	<0.001	0.159	0.010	[0.140,0.179]	<0.001	0.162	0.010	[0.142,0.181]	<0.001
Round 4	0.225	0.010	[0.204,0.245]	<0.001	0.216	0.010	[0.195,0.236]	<0.001	0.219	0.010	[0.198,0.239]	<0.001
Round 5	0.243	0.011	[0.221,0.264]	<0.001	0.236	0.011	[0.215,0.257]	<0.001	0.241	0.011	[0.220,0.262]	<0.001
**Emergency room visits (ref: none)**												
One					0.016	0.015	[-0.013,0.045]	0.27	0.015	0.015	[-0.013,0.044]	0.30
Two					0.074	0.031	[0.014,0.134]	0.02	0.069	0.030	[0.010,0.129]	0.02
Three					-0.012	0.061	[-0.131,0.107]	0.84	-0.025	0.060	[-0.142,0.092]	0.67
Four					0.074	0.092	[-0.107,0.255]	0.43	0.068	0.093	[-0.113,0.250]	0.46
Five or more					0.050	0.091	[-0.129,0.230]	0.58	0.043	0.094	[-0.140,0.227]	0.65
**Hospitalizations (ref: none)**												
One					0.075	0.017	[0.041,0.108]	<0.001	0.075	0.017	[0.042,0.109]	<0.001
Two					0.087	0.037	[0.016,0.159]	0.02	0.086	0.036	[0.015,0.157]	0.02
Three					0.231	0.070	[0.095,0.367]	0.001	0.247	0.069	[0.112,0.382]	<0.001
Four					0.284	0.093	[0.102,0.465]	0.002	0.295	0.091	[0.116,0.473]	0.001
Five or more					0.235	0.160	[-0.079,0.548]	0.14	0.255	0.164	[-0.066,0.576]	0.12
**Number of nights in hospital**					-0.002	0.001	(-0.004,0.001)	0.14	0.002	0.001	[0.001,0.004]	0.006
**Unemployed at interview date**					-0.123	0.022	(-0.167,-0.079)	<0.001	0.134	0.020	[0.094,0.174]	<0.001
**Family members with fair/poor self-rated physical health (ref: zero)**												
One									0.072	0.011	[0.051,0.094]	<0.001
Two									0.177	0.022	[0.134,0.220]	<0.001
Three									0.288	0.045	[0.200,0.377]	<0.001
Four									0.261	0.107	[0.051,0.471]	0.02
Five or more									0.403	0.167	[0.076,0.730]	0.02
**Family members with fair/poor self-rated mental health (ref: zero)**												
One									0.242	0.012	[0.219,0.266]	<0.001
Two									0.451	0.028	[0.397,0.506]	<0.001
Three									0.546	0.059	[0.430,0.662]	<0.001
Four									0.703	0.161	[0.387,1.018]	<0.001
Five or more									0.844	0.289	[0.278,1.409]	0.003
**1+ family members went to emergency room (vs. none)**									0.009	0.011	[-0.012,0.031]	0.38
**1+ family members hospitalized (vs. none)**									-0.005	0.012	[-0.029,0.018]	0.65
**Someone in household employed (vs. no one)**									-0.033	0.022	[-0.076,0.010]	0.14
Constant	2.526	0.006	[2.513,2.538]	<0.001	2.428	0.015	[2.399,2.458]	<0.001	2.335	0.026	[2.283,2.386]	<0.001
R^2	0.016				0.020				0.041			

© 2021 Unger ES et al. *JAMA Health Forum.*

eTable 5: Event study models: parameter estimates for self-rated mental health over time

Analytic population includes all adults living in households with at least one disabled adult, 1996-2017.

	Unadjusted				Adjusted for individual-level covariates				Adjusted for individual- and household-level covariates			
	Coef.	SE	95% CI	p	Coef.	SE	95% CI	p	Coef.	SE	95% CI	p
**Never disabled adult: Event period (ref: One round pre)**												
Never disabled adult: Four rounds pre	0.010	0.085	(-0.156,0.177)	0.90	0.015	0.085	(-0.152,0.182)	0.86	0.002	0.084	(-0.163,0.167)	0.98
Never disabled adult: Three rounds pre	-0.063	0.062	(-0.184,0.058)	0.31	-0.064	0.062	(-0.185,0.056)	0.30	-0.092	0.061	(-0.212,0.027)	0.13
Never disabled adult: Two rounds pre	0.020	0.042	(-0.063,0.102)	0.64	0.022	0.042	(-0.061,0.104)	0.60	0.013	0.042	(-0.069,0.095)	0.75
Never disabled adult: One round post	0.054	0.038	(-0.020,0.128)	0.15	0.052	0.038	(-0.022,0.126)	0.16	0.063	0.037	(-0.010,0.137)	0.09
Never disabled adult: Two rounds post	0.069	0.041	(-0.012,0.149)	0.09	0.067	0.041	(-0.013,0.148)	0.10	0.070	0.041	(-0.009,0.150)	0.08
Never disabled adult: Three rounds post	0.054	0.046	(-0.037,0.145)	0.25	0.051	0.046	(-0.040,0.142)	0.27	0.027	0.046	(-0.063,0.117)	0.56
Never disabled adult: Four rounds post	0.117	0.065	(-0.010,0.244)	0.07	0.112	0.065	(-0.015,0.239)	0.08	0.069	0.064	(-0.057,0.196)	0.28
**Ever disabled adult: Event period (ref: One round pre)**												
Ever disabled adult: Four rounds pre	0.024	0.285	(-0.535,0.583)	0.93	0.028	0.285	(-0.530,0.586)	0.92	-0.027	0.301	(-0.618,0.563)	0.93
Ever disabled adult: Three rounds pre	0.115	0.151	(-0.181,0.411)	0.45	0.104	0.147	(-0.183,0.391)	0.48	0.076	0.146	(-0.209,0.362)	0.60
Ever disabled adult: Two rounds pre	-0.017	0.118	(-0.248,0.214)	0.89	-0.032	0.117	(-0.262,0.197)	0.78	-0.054	0.116	(-0.281,0.173)	0.64
Ever disabled adult: One round post	-0.122	0.119	(-0.355,0.111)	0.31	-0.118	0.119	(-0.351,0.115)	0.32	-0.118	0.116	(-0.345,0.109)	0.31
Ever disabled adult: Two rounds post	-0.171	0.116	(-0.398,0.055)	0.14	-0.168	0.116	(-0.396,0.060)	0.15	-0.163	0.117	(-0.393,0.067)	0.16
Ever disabled adult: Three rounds post	-0.027	0.109	(-0.241,0.187)	0.81	-0.031	0.109	(-0.245,0.182)	0.77	-0.037	0.109	(-0.251,0.177)	0.73
Ever disabled adult: Four rounds post	-0.318	0.149	(-0.610,-0.027)	0.03	-0.305	0.146	(-0.592,-0.018)	0.04	-0.295	0.146	(-0.582,-0.009)	0.04
**Medicaid home care recipient: Event period (ref: One round pre)**												
Medicaid home care recipient: Four rounds pre	-0.051	0.101	(-0.249,0.148)	0.62	-0.066	0.101	(-0.264,0.132)	0.52	-0.066	0.100	(-0.262,0.130)	0.51
Medicaid home care recipient: Three rounds pre	0.012	0.063	(-0.112,0.135)	0.86	0.005	0.062	(-0.117,0.127)	0.94	-0.004	0.062	(-0.125,0.117)	0.95
Medicaid home care recipient: Two rounds pre	0.067	0.045	(-0.021,0.154)	0.14	0.060	0.045	(-0.027,0.148)	0.18	0.062	0.044	(-0.025,0.149)	0.16
Medicaid home care recipient: One round post	-0.045	0.042	(-0.126,0.037)	0.28	-0.020	0.042	(-0.102,0.061)	0.63	-0.021	0.041	(-0.102,0.060)	0.61
Medicaid home care recipient: Two rounds post	-0.014	0.044	(-0.100,0.072)	0.75	-0.013	0.044	(-0.099,0.072)	0.76	-0.003	0.043	(-0.087,0.081)	0.94
Medicaid home care recipient: Three rounds post	-0.002	0.056	(-0.111,0.107)	0.97	-0.006	0.055	(-0.115,0.102)	0.91	0.002	0.054	(-0.105,0.108)	0.98
Medicaid home care recipient: Four rounds post	0.053	0.078	(-0.099,0.205)	0.50	0.047	0.078	(-0.106,0.199)	0.55	0.047	0.077	(-0.104,0.197)	0.54
**Round (ref: 1)**												
Round 2	-0.097	0.008	(-0.112,-0.082)	<0.001	-0.092	0.008	(-0.107,-0.076)	<0.001	-0.091	0.008	(-0.106,-0.076)	<0.001
Round 3	-0.135	0.008	(-0.151,-0.119)	<0.001	-0.128	0.008	(-0.144,-0.112)	<0.001	-0.130	0.008	(-0.146,-0.115)	<0.001
Round 4	-0.179	0.008	(-0.195,-0.162)	<0.001	-0.171	0.008	(-0.188,-0.155)	<0.001	-0.175	0.008	(-0.192,-0.159)	<0.001
Round 5	-0.197	0.009	(-0.214,-0.179)	<0.001	-0.191	0.009	(-0.209,-0.174)	<0.001	-0.196	0.009	(-0.213,-0.179)	<0.001
**Emergency room visits (ref: none)**												

© 2021 Unger ES et al. *JAMA Health Forum.*

	Unadjusted				Adjusted for individual-level covariates				Adjusted for individual- and household-level covariates			
	Coef.	SE	95% CI	p	Coef.	SE	95% CI	p	Coef.	SE	95% CI	p
One					-0.015	0.012	(-0.038,0.007)	0.18	-0.014	0.011	(-0.036,0.009)	0.23
Two					-0.062	0.024	(-0.109,-0.015)	0.01	-0.057	0.024	(-0.103,-0.010)	0.02
Three					-0.004	0.048	(-0.098,0.090)	0.94	0.004	0.047	(-0.089,0.097)	0.93
Four					-0.078	0.071	(-0.218,0.062)	0.28	-0.069	0.071	(-0.209,0.071)	0.33
Five or more					-0.039	0.073	(-0.182,0.104)	0.60	-0.028	0.075	(-0.175,0.118)	0.70
**Hospitalizations (ref: none)**												
One					-0.064	0.014	(-0.091,-0.037)	<0.001	-0.065	0.013	(-0.091,-0.039)	<0.001
Two					-0.074	0.029	(-0.131,-0.017)	0.01	-0.073	0.029	(-0.129,-0.017)	0.01
Three					-0.184	0.055	(-0.292,-0.077)	0.001	-0.196	0.054	(-0.302,-0.089)	<0.001
Four					-0.192	0.075	(-0.338,-0.046)	0.01	-0.200	0.074	(-0.344,-0.055)	0.007
Five or more					-0.268	0.118	(-0.500,-0.036)	0.02	-0.285	0.121	(-0.522,-0.047)	0.02
**Number of nights in hospital**					-0.002	0.001	(-0.003,-0.001)	0.003	-0.002	0.001	(-0.003,-0.001)	0.003
**Unemployed at interview date**					-0.112	0.016	(-0.144,-0.080)	<0.001	-0.096	0.019	(-0.132,-0.060)	<0.001
**Family members with fair/poor self-rated physical health (ref: zero)**												
One									-0.060	0.009	(-0.077,-0.043)	<0.001
Two									-0.150	0.017	(-0.184,-0.116)	<0.001
Three									-0.226	0.036	(-0.296,-0.156)	<0.001
Four									-0.299	0.082	(-0.460,-0.139)	<0.001
Five or more									-0.324	0.135	(-0.588,-0.060)	0.02
**Family members with fair/poor self-rated mental health (ref: zero)**												
One									-0.200	0.009	(-0.218,-0.182)	<0.001
Two									-0.372	0.022	(-0.415,-0.329)	<0.001
Three									-0.448	0.046	(-0.537,-0.358)	<0.001
Four									-0.532	0.127	(-0.782,-0.282)	<0.001
Five or more									-0.688	0.237	(-1.152,-0.224)	0.004
**1+ family members went to emergency room (vs. none)**									-0.007	0.009	(-0.024,0.010)	0.41
**1+ family members hospitalized (vs. none)**									0.004	0.009	(-0.015,0.022)	0.69
**Someone in household employed (vs. no one)**									0.023	0.018	(-0.011,0.057)	0.19
Constant	-0.013	0.006	(-0.024,-0.001)	0.034	0.069	0.013	(0.045,0.094)	<0.001	0.153	0.021	(0.112,0.194)	<0.001
R^2	0.015				0.019				0.040			
p for parallel pre-trends	0.56				0.52				0.35			

© 2021 Unger ES et al. *JAMA Health Forum.*

eTable 6: Event study models: parameter estimates for self-rated physical health over time
Analytic population includes all adults living in households with at least one disabled adult, 1996-2017.

	Unadjusted				Adjusted for individual-level covariates				Adjusted for individual- and household-level covariates			
	Coef.	SE	95% CI	p	Coef.	SE	95% CI	p	Coef.	SE	95% CI	p
**Never disabled adult: Event period (ref: One round pre)**												
Never disabled adult: Four rounds pre	-0.017	0.082	(-0.178,0.144)	0.83	-0.008	0.084	(-0.172,0.155)	0.92	-0.020	0.079	(-0.175,0.136)	0.81
Never disabled adult: Three rounds pre	-0.040	0.051	(-0.140,0.060)	0.44	-0.042	0.051	(-0.141,0.057)	0.40	-0.063	0.050	(-0.161,0.035)	0.21
Never disabled adult: Two rounds pre	-0.037	0.035	(-0.106,0.033)	0.30	-0.033	0.035	(-0.102,0.036)	0.35	-0.035	0.035	(-0.104,0.033)	0.31
Never disabled adult: One round post	-0.017	0.034	(-0.084,0.051)	0.64	-0.020	0.034	(-0.087,0.048)	0.57	-0.019	0.034	(-0.085,0.048)	0.59
Never disabled adult: Two rounds post	0.011	0.038	(-0.062,0.085)	0.76	0.010	0.037	(-0.063,0.083)	0.79	0.003	0.038	(-0.070,0.077)	0.94
Never disabled adult: Three rounds post	0.050	0.043	(-0.034,0.134)	0.25	0.045	0.043	(-0.039,0.129)	0.29	0.024	0.042	(-0.058,0.106)	0.57
Never disabled adult: Four rounds post	0.002	0.056	(-0.108,0.112)	0.97	-0.007	0.056	(-0.117,0.103)	0.90	-0.042	0.055	(-0.150,0.066)	0.44
**Ever disabled adult: Event period (ref: One round pre)**												
Ever disabled adult: Four rounds pre	-0.061	0.242	(-0.536,0.413)	0.80	-0.047	0.251	(-0.538,0.444)	0.85	-0.072	0.249	(-0.559,0.416)	0.77
Ever disabled adult: Three rounds pre	0.033	0.165	(-0.291,0.357)	0.84	0.006	0.155	(-0.298,0.310)	0.97	-0.011	0.151	(-0.307,0.284)	0.94
Ever disabled adult: Two rounds pre	-0.041	0.109	(-0.255,0.174)	0.71	-0.071	0.105	(-0.276,0.135)	0.50	-0.080	0.102	(-0.280,0.120)	0.43
Ever disabled adult: One round post	0.044	0.084	(-0.121,0.209)	0.60	0.054	0.080	(-0.103,0.210)	0.50	0.059	0.079	(-0.097,0.214)	0.46
Ever disabled adult: Two rounds post	-0.098	0.092	(-0.278,0.082)	0.29	-0.092	0.091	(-0.270,0.086)	0.31	-0.092	0.092	(-0.272,0.088)	0.32
Ever disabled adult: Three rounds post	0.062	0.105	(-0.144,0.267)	0.56	0.051	0.101	(-0.147,0.248)	0.62	0.044	0.101	(-0.155,0.243)	0.66
Ever disabled adult: Four rounds post	0.203	0.175	(-0.141,0.547)	0.25	0.227	0.165	(-0.097,0.551)	0.17	0.219	0.159	(-0.094,0.531)	0.17
**Medicaid home care recipient: Event period (ref: One round pre)**												
Medicaid home care recipient: Four rounds pre	0.071	0.091	(-0.107,0.249)	0.44	0.036	0.088	(-0.137,0.209)	0.69	0.048	0.086	(-0.121,0.218)	0.58
Medicaid home care recipient: Three rounds pre	0.087	0.056	(-0.023,0.196)	0.12	0.066	0.055	(-0.041,0.174)	0.23	0.066	0.055	(-0.041,0.173)	0.23
Medicaid home care recipient: Two rounds pre	0.022	0.036	(-0.048,0.091)	0.54	0.010	0.036	(-0.060,0.080)	0.78	0.012	0.036	(-0.058,0.082)	0.74
Medicaid home care recipient: One round post	-0.078	0.034	(-0.144,-0.012)	0.02	-0.028	0.034	(-0.094,0.039)	0.41	-0.024	0.034	(-0.090,0.042)	0.47
Medicaid home care recipient: Two rounds post	0.014	0.038	(-0.059,0.088)	0.70	0.015	0.037	(-0.057,0.088)	0.68	0.023	0.037	(-0.049,0.095)	0.53
Medicaid home care recipient: Three rounds post	0.014	0.042	(-0.068,0.096)	0.74	0.009	0.041	(-0.073,0.090)	0.83	0.014	0.041	(-0.066,0.094)	0.73
Medicaid home care recipient: Four rounds post	0.061	0.062	(-0.061,0.183)	0.33	0.049	0.062	(-0.073,0.171)	0.43	0.045	0.062	(-0.076,0.166)	0.47
**Round (ref: 1)**												
Round 2	-0.009	0.007	(-0.023,0.004)	0.19	0.003	0.007	(-0.010,0.017)	0.62	-0.002	0.007	(-0.015,0.012)	0.78
Round 3	-0.028	0.007	(-0.042,-0.014)	<0.001	-0.012	0.007	(-0.026,0.002)	0.09	-0.021	0.007	(-0.035,-0.007)	0.004
Round 4	-0.053	0.008	(-0.068,-0.038)	<0.001	-0.037	0.008	(-0.052,-0.022)	<0.001	-0.048	0.008	(-0.063,-0.033)	<0.001
Round 5	-0.057	0.008	(-0.072,-0.041)	<0.001	-0.047	0.008	(-0.063,-0.032)	<0.001	-0.059	0.008	(-0.074,-0.044)	<0.001
**Emergency room visits (ref: none)**												

© 2021 Unger ES et al. *JAMA Health Forum.*

	Unadjusted				Adjusted for individual-level covariates				Adjusted for individual- and household-level covariates			
	Coef.	SE	95% CI	p	Coef.	SE	95% CI	p	Coef.	SE	95% CI	p
One					-0.096	0.010	(-0.115,-0.076)	<0.001	-0.094	0.010	(-0.114,-0.075)	<0.001
Two					-0.171	0.020	(-0.211,-0.131)	<0.001	-0.167	0.020	(-0.207,-0.127)	<0.001
Three					-0.170	0.040	(-0.248,-0.092)	<0.001	-0.162	0.039	(-0.239,-0.084)	<0.001
Four					-0.240	0.059	(-0.355,-0.125)	<0.001	-0.238	0.059	(-0.353,-0.123)	<0.001
Five or more					-0.212	0.063	(-0.336,-0.088)	0.001	-0.199	0.062	(-0.321,-0.077)	0.001
**Hospitalizations (ref: none)**												
One					-0.142	0.012	(-0.165,-0.118)	<0.001	-0.144	0.012	(-0.167,-0.120)	<0.001
Two					-0.171	0.025	(-0.219,-0.123)	<0.001	-0.174	0.025	(-0.222,-0.126)	<0.001
Three					-0.241	0.044	(-0.328,-0.154)	<0.001	-0.255	0.044	(-0.342,-0.168)	<0.001
Four					-0.293	0.060	(-0.410,-0.175)	<0.001	-0.292	0.060	(-0.410,-0.175)	<0.001
Five or more					-0.400	0.108	(-0.610,-0.189)	<0.001	-0.408	0.107	(-0.618,-0.198)	<0.001
**Number of nights in hospital**					-0.003	0.001	(-0.004,-0.002)	<0.001	-0.003	0.001	(-0.004,-0.002)	<0.001
**Unemployed at interview date**					-0.136	0.016	(-0.167,-0.104)	<0.001	-0.129	0.018	(-0.164,-0.093)	<0.001
**Family members with fair/poor self-rated physical health (ref: zero)**												
One									-0.148	0.008	(-0.163,-0.133)	<0.001
Two									-0.257	0.016	(-0.288,-0.226)	<0.001
Three									-0.295	0.036	(-0.365,-0.225)	<0.001
Four									-0.442	0.078	(-0.595,-0.289)	<0.001
Five or more									-0.762	0.105	(-0.967,-0.557)	<0.001
**Family members with fair/poor self-rated mental health (ref: zero)**												
One									-0.061	0.008	(-0.076,-0.045)	<0.001
Two									-0.155	0.019	(-0.192,-0.118)	<0.001
Three									-0.188	0.045	(-0.276,-0.099)	<0.001
Four									-0.348	0.118	(-0.579,-0.117)	0.003
Five or more									-0.152	0.182	(-0.509,0.205)	0.40
**1+ family members went to emergency room (vs. none)**									0.001	0.008	(-0.014,0.016)	0.92
**1+ family members hospitalized (vs. none)**									0.029	0.008	(0.013,0.046)	0.001
**Someone in household employed (vs. no one)**									0.007	0.016	(-0.024,0.038)	0.67
Constant	-0.167	0.005	(-0.177,-0.157)	<0.001	-0.056	0.012	(-0.079,-0.032)	<0.001	0.042	0.019	(0.004,0.080)	0.03
R^2	0.002				0.021				0.038			
p for parallel pre-trends	0.74				0.76				0.58			

© 2021 Unger ES et al. *JAMA Health Forum.*

eTable 7: Association between Medicaid home care onset and caregivers' self-rated mental health, by demographic variables

Results shown for all likely caregivers interviewed 1996-2017. All estimates are from fully adjusted models.

	Coef.	95% CI for Coef.	Mean pre-onset self-rated mental health	Percent change	95% CI for percent change	p	p for difference
**Interaction with gender**							
Men	0.057	(-0.035, 0.148)	2.192	2.6%	(-1.6%, 6.8%)	0.23	0.58
Women	0.093	(0.001, 0.184)	2.232	4.2%	(0.1%, 8.2%)	0.047	
**Interaction with cognitive impairments**							
No one in household cognitively impaired	0.054	(-0.094, 0.202)	2.401	2.2%	(-3.9%, 8.4%)	0.48	0.75
Someone in household cognitively impaired	0.081	(0.009, 0.153)	2.160	3.7%	(0.4%, 7.1%)	0.028	
**Interaction with employment status**							
Employed	0.045	(-0.042, 0.131)	2.512	1.8%	(-1.7%, 5.2%)	0.31	0.40
Not employed	0.100	(0.005, 0.194)	1.959	5.1%	(0.3%, 9.9%)	0.039	
**Interaction with poverty**							
Above poverty	0.042	(-0.033, 0.117)	2.363	1.8%	(-1.4%, 5.0%)	0.27	0.16
Below/near poverty	0.148	(0.022, 0.273)	1.885	7.8%	(1.2%, 14.5%)	0.021	
**Interaction with age**							
Under age 65	0.094	(0.02, 0.167)	2.258	4.1%	(0.9%, 7.4%)	0.012	0.32
Over age 65	0.014	(-0.123, 0.152)	2.048	0.7%	(-6.0%, 7.4%)	0.84	
**Interaction with race/ethnicity**							
White non-Hispanic	0.048	(-0.07, 0.165)	2.221	2.1%	(-3.2%, 7.4%)	0.43	0.01
Hispanic/Latinx	0.192	(0.07, 0.313)	2.156	8.9%	(3.3%, 14.5%)	0.002	
Black non-Hispanic	0.087	(-0.029, 0.204)	2.213	3.9%	(-1.3%, 9.2%)	0.14	
Asian non-Hispanic	-0.143	(-0.315, 0.029)	2.440	-5.9%	(-12.9%, 1.2%)	0.10	
Multiple race or other	-0.343	(-0.801, 0.116)	1.937	-17.7%	(-41.4%, 6.0%)	0.14	
**Overall**	0.075	(0.01, 0.14)	2.213	3.4%	(0.5%, 6.3%)	0.024	

© 2021 Unger ES et al. *JAMA Health Forum.*

eTable 8: Weighted difference-in-difference models: parameter estimates for self-rated mental health

Analytic population includes all adults living in households with at least one disabled adult, 1996-2017. "Medicaid home care onset: Likely caregiver" is the primary difference-in-difference estimate of interest: the association between onset of Medicaid home care in the household and self-rated mental health among likely caregivers.

	**Unadjusted**				**Adjusted for individual-level covariates**				**Adjusted for individual- and household-level covariates**			
	**Coef.**	**SE**	**95% CI**	**p**	**Coef.**	**SE**	**95% CI**	**p**	**Coef.**	**SE**	**95% CI**	**p**
**Medicaid home care onset: Likely caregiver**	0.010	0.043	(-0.075,0.095)	0.82	0.008	0.043	(-0.077,0.094)	0.85	0.019	0.043	(-0.065,0.103)	0.66
**Medicaid home care onset: Disabled non-recipient**	-0.451	0.227	(-0.895,-0.007)	0.05	-0.421	0.214	(-0.841,-0.002)	0.05	-0.393	0.200	(-0.785,-0.002)	0.05
**Medicaid home care onset: Medicaid home care recipient**	-0.093	0.055	(-0.201,0.016)	0.09	-0.064	0.055	(-0.172,0.044)	0.25	-0.059	0.055	(-0.166,0.048)	0.28
**Round (ref: 1)**												
Round 2	-0.107	0.010	(-0.126,-0.088)	<0.001	-0.101	0.010	(-0.121,-0.082)	<0.001	-0.100	0.010	(-0.119,-0.080)	<0.001
Round 3	-0.156	0.010	(-0.177,-0.136)	<0.001	-0.149	0.010	(-0.169,-0.128)	<0.001	-0.148	0.010	(-0.168,-0.128)	<0.001
Round 4	-0.210	0.011	(-0.230,-0.189)	<0.001	-0.200	0.011	(-0.221,-0.180)	<0.001	-0.201	0.011	(-0.222,-0.180)	<0.001
Round 5	-0.219	0.011	(-0.241,-0.198)	<0.001	-0.212	0.011	(-0.234,-0.191)	<0.001	-0.214	0.011	(-0.235,-0.192)	<0.001
**Emergency room visits (ref: none)**												
One					-0.007	0.015	(-0.037,0.022)	0.62	-0.006	0.015	(-0.036,0.023)	0.67
Two					-0.081	0.031	(-0.142,-0.019)	0.01	-0.075	0.031	(-0.136,-0.013)	0.02
Three					-0.068	0.062	(-0.190,0.053)	0.27	-0.061	0.062	(-0.182,0.060)	0.32
Four					-0.029	0.081	(-0.187,0.129)	0.72	-0.031	0.081	(-0.189,0.127)	0.70
Five or more					0.089	0.113	(-0.133,0.311)	0.43	0.095	0.114	(-0.129,0.318)	0.41
**Hospitalizations (ref: none)**												
One					-0.065	0.018	(-0.101,-0.029)	<0.001	-0.065	0.018	(-0.101,-0.029)	<0.001
Two					-0.085	0.041	(-0.165,-0.004)	0.04	-0.082	0.041	(-0.162,-0.002)	0.05
Three					-0.192	0.077	(-0.343,-0.040)	0.01	-0.207	0.077	(-0.359,-0.056)	0.007
Four					-0.190	0.101	(-0.387,0.007)	0.06	-0.194	0.099	(-0.387,0.000)	0.05
Five or more					-0.174	0.152	(-0.473,0.124)	0.25	-0.174	0.155	(-0.477,0.130)	0.26
**Number of nights in hospital**					-0.002	0.001	(-0.004,0.001)	0.14	-0.002	0.001	(-0.004,0.001)	0.16
**Unemployed at interview date**					-0.123	0.022	(-0.167,-0.079)	<0.001	-0.117	0.025	(-0.166,-0.068)	<0.001
**Family members with fair/poor self-rated physical health (ref: zero)**												
One									-0.051	0.011	(-0.074,-0.029)	<0.001
Two									-0.124	0.023	(-0.170,-0.079)	<0.001
Three									-0.181	0.049	(-0.277,-0.084)	<0.001
Four									-0.174	0.083	(-0.338,-0.011)	0.04
Five or more									-0.106	0.166	(-0.432,0.219)	0.52
**Family members with fair/poor self-rated mental health (ref: zero)**												
One									-0.203	0.012	(-0.227,-0.179)	<0.001
Two									-0.324	0.029	(-0.380,-0.268)	<0.001
Three									-0.310	0.067	(-0.441,-0.180)	<0.001
Four									-0.899	0.278	(-1.444,-0.353)	0.001
Five or more									-0.864	0.336	(-1.523,-0.206)	0.01
**1+ family members went to emergency room (vs. none)**									-0.005	0.011	(-0.027,0.018)	0.69
**1+ family members hospitalized (vs. none)**									-0.008	0.013	(-0.033,0.016)	0.51
**Someone in household employed (vs. no one)**									0.006	0.023	(-0.040,0.052)	0.78
Constant	0.043	0.006	(0.031,0.056)	<0.001	0.130	0.016	(0.098,0.163)	<0.001	0.211	0.027	(0.158,0.264)	<0.001
R^2	0.02				0.024				0.041			

© 2021 Unger ES et al. *JAMA Health Forum.*

eTable 9: Weighted difference-in-difference models: parameter estimates for self-rated physical health

Analytic population includes all adults living in households with at least one disabled adult, 1996-2017. "Medicaid home care onset: Likely caregiver" is the primary difference-in-difference estimate of interest: the association between onset of Medicaid home care in the household and self-rated mental health among likely caregivers.

	**Unadjusted**				**Adjusted for individual-level covariates**				**Adjusted for individual- and household-level covariates**			
	Coef.	SE	95% CI	p	**Coef.**	**SE**	**95% CI**	**p**	**Coef.**	**SE**	**95% CI**	**p**
**Medicaid home care onset: Likely caregiver**	-0.034	0.038	(-0.109,0.041)	0.38	-0.037	0.038	(-0.112,0.037)	0.32	-0.036	0.037	(-0.109,0.037)	0.34
**Medicaid home care onset: Disabled non-recipient**	-0.043	0.160	(-0.356,0.270)	0.79	0.036	0.133	(-0.225,0.297)	0.79	0.055	0.124	(-0.187,0.298)	0.66
**Medicaid home care onset: Medicaid home care recipient**	-0.128	0.044	(-0.214,-0.043)	0.003	-0.069	0.044	(-0.156,0.018)	0.12	-0.065	0.044	(-0.151,0.022)	0.14
**Round (ref: 1)**												
Round 2	-0.018	0.009	(-0.036,-0.001)	0.04	-0.003	0.009	(-0.021,0.014)	0.73	-0.008	0.009	(-0.026,0.009)	0.34
Round 3	-0.039	0.009	(-0.058,-0.021)	<0.001	-0.021	0.009	(-0.039,-0.003)	0.03	-0.029	0.009	(-0.047,-0.010)	0.002
Round 4	-0.080	0.010	(-0.100,-0.061)	<0.001	-0.060	0.010	(-0.079,-0.041)	<0.001	-0.069	0.010	(-0.088,-0.050)	<0.001
Round 5	-0.084	0.010	(-0.104,-0.064)	<0.001	-0.070	0.010	(-0.090,-0.049)	<0.001	-0.079	0.010	(-0.099,-0.059)	<0.001
**Emergency room visits (ref: none)**												
One					-0.085	0.014	(-0.112,-0.058)	<0.001	-0.085	0.014	(-0.112,-0.058)	<0.001
Two					-0.163	0.028	(-0.219,-0.107)	<0.001	-0.161	0.028	(-0.217,-0.106)	<0.001
Three					-0.236	0.053	(-0.339,-0.132)	<0.001	-0.233	0.053	(-0.336,-0.129)	<0.001
Four					-0.144	0.079	(-0.298,0.011)	0.07	-0.155	0.078	(-0.308,-0.003)	0.05
Five or more					-0.185	0.092	(-0.365,-0.006)	0.04	-0.179	0.094	(-0.363,0.006)	0.06
**Hospitalizations (ref: none)**												
One					-0.158	0.017	(-0.191,-0.125)	<0.001	-0.158	0.017	(-0.191,-0.126)	<0.001
Two					-0.193	0.036	(-0.264,-0.123)	<0.001	-0.194	0.036	(-0.264,-0.124)	<0.001
Three					-0.236	0.061	(-0.356,-0.115)	<0.001	-0.250	0.061	(-0.369,-0.131)	<0.001
Four					-0.358	0.081	(-0.517,-0.199)	<0.001	-0.350	0.082	(-0.511,-0.190)	<0.001
Five or more					-0.518	0.135	(-0.782,-0.253)	<0.001	-0.508	0.136	(-0.775,-0.240)	<0.001
**Number of nights in hospital**					-0.003	0.001	(-0.005,-0.001)	0.002	-0.003	0.001	(-0.005,-0.001)	0.002
**Unemployed at interview date**					-0.143	0.022	(-0.187,-0.100)	<0.001	-0.130	0.026	(-0.181,-0.080)	<0.001
**Family members with fair/poor self-rated physical health (ref: zero)**												
One									-0.138	0.010	(-0.157,-0.118)	<0.001
Two									-0.231	0.020	(-0.270,-0.192)	<0.001
Three									-0.265	0.047	(-0.356,-0.173)	<0.001
Four									-0.037	0.122	(-0.277,0.203)	0.76
Five or more									-0.795	0.132	(-1.054,-0.535)	<0.001
**Family members with fair/poor self-rated mental health (ref: zero)**												
One									-0.055	0.010	(-0.076,-0.035)	<0.001
Two									-0.140	0.025	(-0.189,-0.090)	<0.001
Three									-0.157	0.064	(-0.283,-0.031)	0.02
Four									-0.642	0.281	(-1.194,-0.091)	0.02
Five or more									-0.102	0.256	(-0.603,0.399)	0.69
**1+ family members went to emergency room (vs. none)**									0.009	0.010	(-0.011,0.029)	0.38
**1+ family members hospitalized (vs. none)**									0.026	0.011	(0.004,0.049)	0.02
**Someone in household employed (vs. no one)**									0.020	0.023	(-0.026,0.066)	0.40
Constant	-0.094	0.006	(-0.106,-0.082)	<0.001	0.019	0.016	(-0.013,0.051)	0.24	0.084	0.028	(0.029,0.139)	0.003
R^2	0.005				0.025				0.038			

© 2021 Unger ES et al. *JAMA Health Forum.*

eTable 10: Comparison of demographic and health characteristics of study participants with and without high survey weights

All cells show n (percent). Population comprises all adults living in households with at least one disabled adult interviewed in 1996-2017.

	Weight in bottom 90%	Weight in top 10%	p
	N=12,466	N=1,547	
**Someone in household ever receives Medicaid home care**	1,927 (15.5%)	124 (8.0%)	<0.001
**Household role, all rounds**			
Never disabled adult	6,495 (52.1%)	737 (47.6%)	<0.001
Ever disabled adult	5,064 (40.6%)	751 (48.5%)	
Medicaid home care recipient	907 (7.3%)	59 (3.8%)	
**Age at baseline**			
Under age 21	235 (1.9%)	17 (1.1%)	<0.001
Age 21-34	1,565 (12.6%)	146 (9.4%)	
Age 35-49	2,665 (21.4%)	279 (18.0%)	
Age 50-64	3,475 (27.9%)	395 (25.5%)	
Age 65-79	2,978 (23.9%)	432 (27.9%)	
Age 80+	1,548 (12.4%)	278 (18.0%)	
**Gender**			
Male	5,427 (43.5%)	760 (49.1%)	<0.001
Female	7,039 (56.5%)	787 (50.9%)	
**Race/ethnicity**			
White non-Hispanic	5,621 (45.1%)	1,347 (87.1%)	<0.001
Hispanic/Latinx	3,102 (24.9%)	39 (2.5%)	
Black non-Hispanic	2,757 (22.1%)	87 (5.6%)	
Asian non-Hispanic	685 (5.5%)	52 (3.4%)	
Multiple race or other	301 (2.4%)	22 (1.4%)	
**Education level**			
Less than or equal to 8th grade	2,390 (19.4%)	101 (6.6%)	<0.001
9-12 grade, no diploma	1,954 (15.8%)	156 (10.2%)	
High school diploma or GED	4,331 (35.1%)	580 (37.8%)	
Some college or associate's degree	2,178 (17.6%)	369 (24.1%)	
Bachelor's degree	1,033 (8.4%)	192 (12.5%)	
Graduate school	465 (3.8%)	136 (8.9%)	
**Household income level at baseline**			
Poor/negative	2,555 (20.5%)	126 (8.1%)	<0.001
Near poor	1,003 (8.0%)	67 (4.3%)	
Low income	2,545 (20.4%)	250 (16.2%)	
Middle income	3,744 (30.0%)	546 (35.3%)	
High income	2,619 (21.0%)	558 (36.1%)	
**Employed at baseline**	3,884 (31.2%)	528 (34.1%)	0.02
**Someone else in the household ever has cognitive limitations**	5,717 (45.9%)	620 (40.1%)	<0.001
**Self-rated physical health at baseline**			
Excellent	1,617 (13.0%)	221 (14.3%)	<0.001
Very good	2,223 (17.8%)	361 (23.3%)	
Good	3,300 (26.5%)	403 (26.1%)	
Fair	3,002 (24.1%)	313 (20.2%)	
Poor	2,324 (18.6%)	249 (16.1%)	
**Self-rated mental health at baseline**			
Excellent	3,041 (24.4%)	402 (26.0%)	0.006
Very good	2,771 (22.2%)	395 (25.5%)	
Good	3,837 (30.8%)	439 (28.4%)	
Fair	1,874 (15.0%)	207 (13.4%)	
Poor	943 (7.6%)	104 (6.7%)	

© 2021 Unger ES et al. *JAMA Health Forum.*

eTable 11: Association between self-rated mental health and clinically interpretable measures of mental health disorders

Analytic population includes all adults living in households with at least one disabled adult and with data available for the Patient Health Questionnaire-2 (PHQ-2) and Kessler-6, 2004-2017.

	Coefficient	95% CI	p
**Outcome: Depression (PHQ-2 score ≥ 3)**			
1% of a standard deviation improvement in self-rated mental health	-0.0110	(-0.0115, -0.0105)	<0.001
Constant	-1.611	(-1.664, -1.557)	<0.001
Pseudo R^2	0.155		
**Outcome: Severe psychological distress (Kessler-6 score ≥ 13)**			
1% of a standard deviation improvement in self-rated mental health	-0.0126	(-0.0132, -0.0119)	<0.001
Constant	-2.334	(-2.408, -2.261)	<0.001
Pseudo R^2	0.181		

© 2021 Unger ES et al. *JAMA Health Forum.*

eFigures

eFigure 1: Exposure status over time among Medicaid home care recipients

Population comprises all adults interviewed in 1996-2017 who received Medicaid home care in at least one wave. Color blocks indicate the proportion of individuals with each combination of exposure status over time.

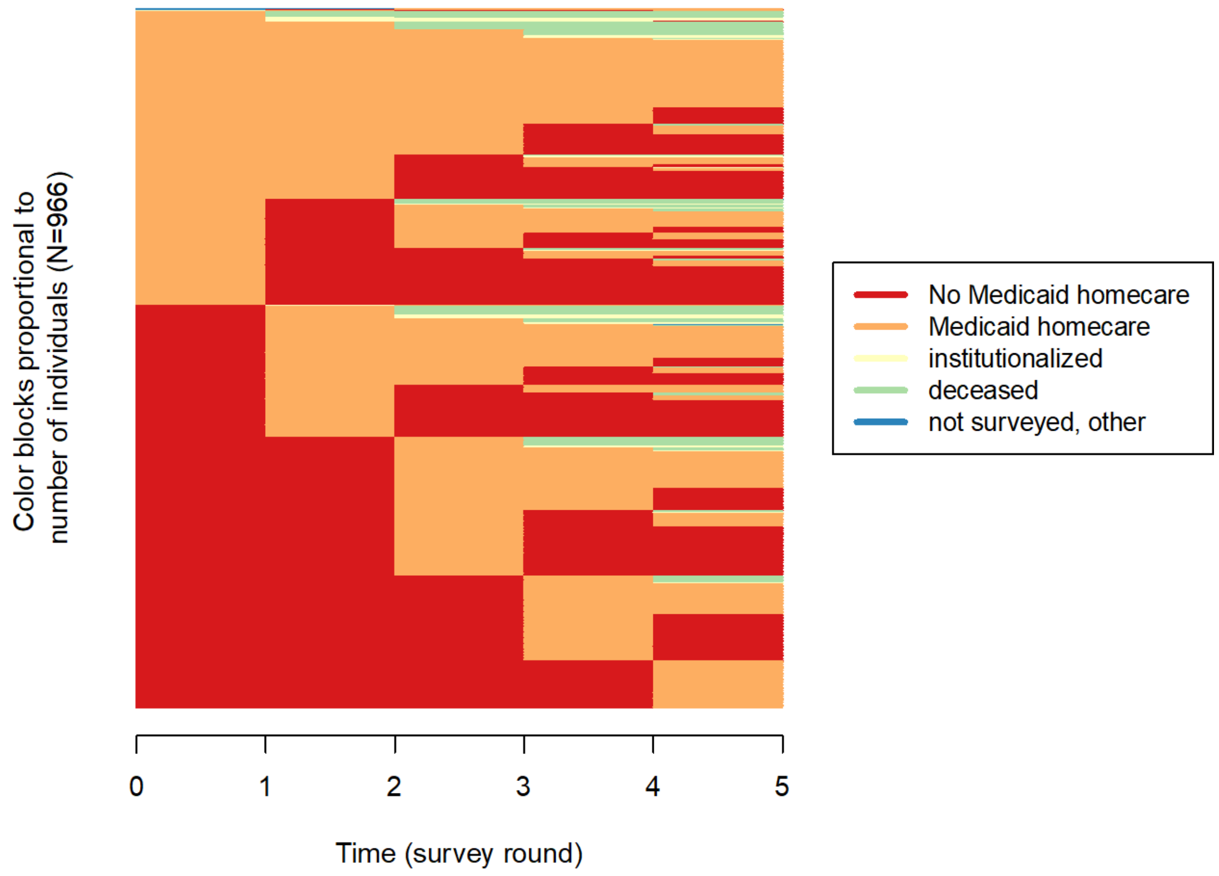

© 2021 Unger ES et al. *JAMA Health Forum.*

eFigure 2: Exposure status over time among all individuals in households ever exposed to Medicaid home care

Population comprises all adults interviewed in 1996-2017 who lived in households in which someone received Medicaid home care in at least one wave. Color blocks indicate the proportion of individuals with each combination of exposure status over time.

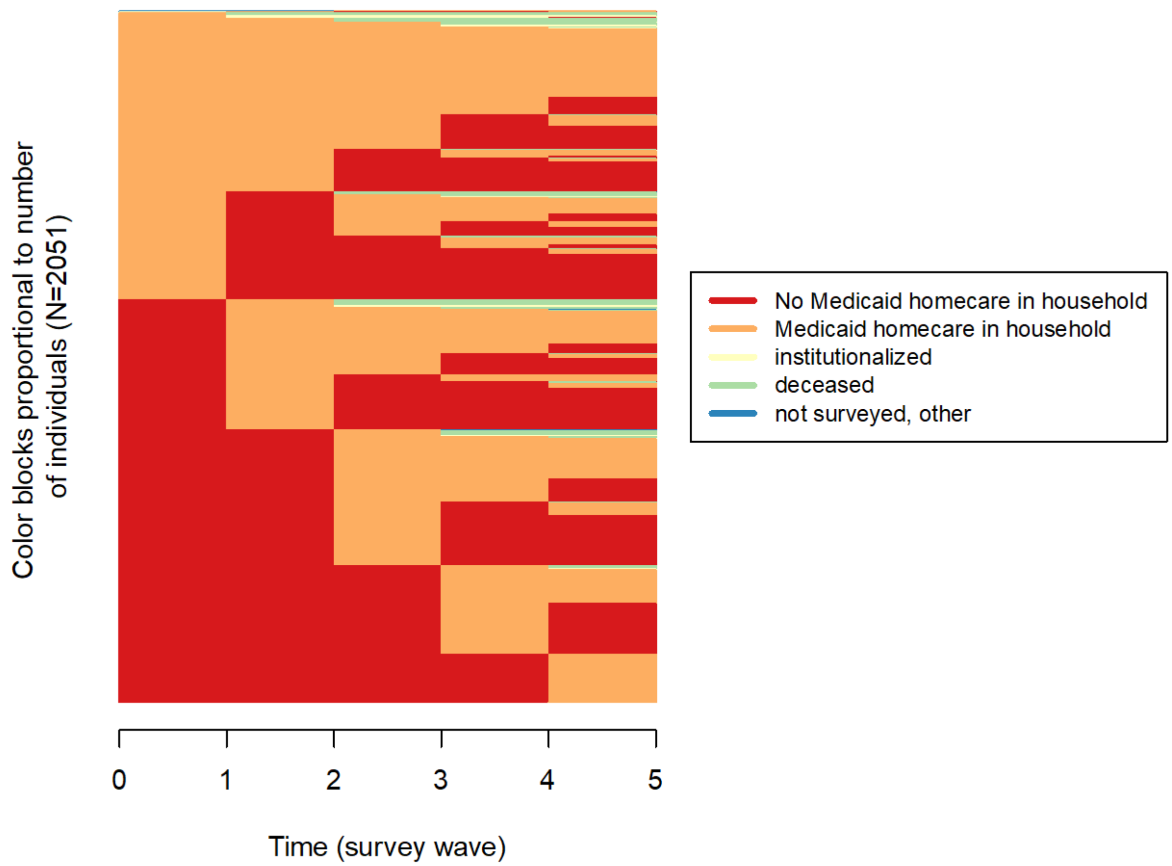

© 2021 Unger ES et al. *JAMA Health Forum.*

eFigure 3: Self-rated health over time relative to onset of Medicaid home care, by household role

Results shown for all adults living in households with at least one disabled adult,1996-2017. Change in self-rated health over time is relative to the round before onset of Medicaid home care, above and beyond time trends in health observed in the full population. Negative numbers on the x-axis indicate rounds prior to onset of Medicaid home care; positive numbers indicate rounds after onset. Rounds are approximately six months apart. Error bars indicate 95% confidence intervals.

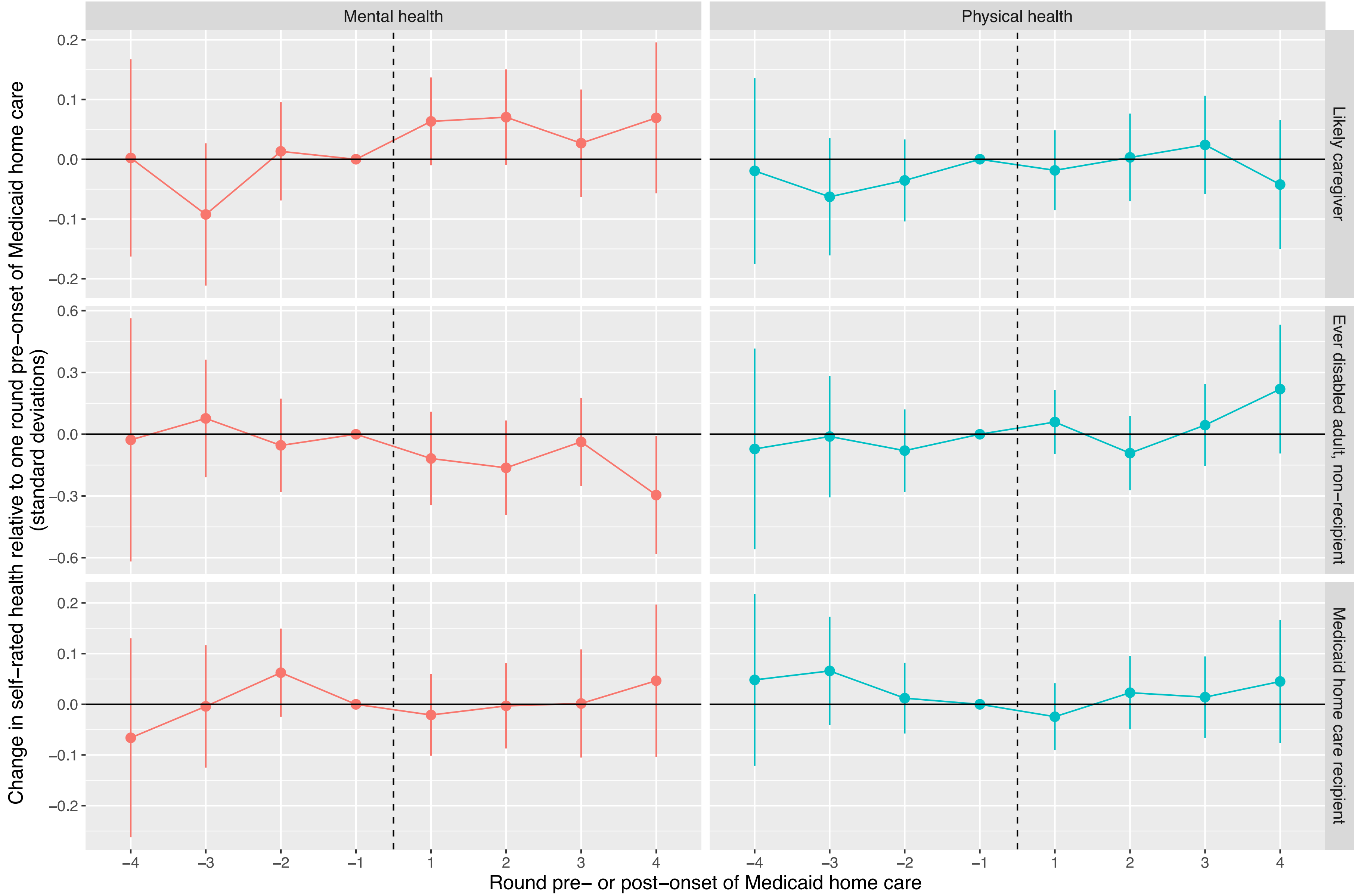

© 2021 Unger ES et al. *JAMA Health Forum.*

eFigure 4: Histogram of the longitudinal weights

Distribution of the longitudinal weights is shown for all adults living in households with at least one disabled adult, 1996-2017.

min	25th pctl	50th pctl	75th pctl	90th pctl	95th pctl	99th pctl	max
918.46	8,160.64	14,087.64	23,661.40	35,436.57	44,738.15	69,114.58	170,898.9

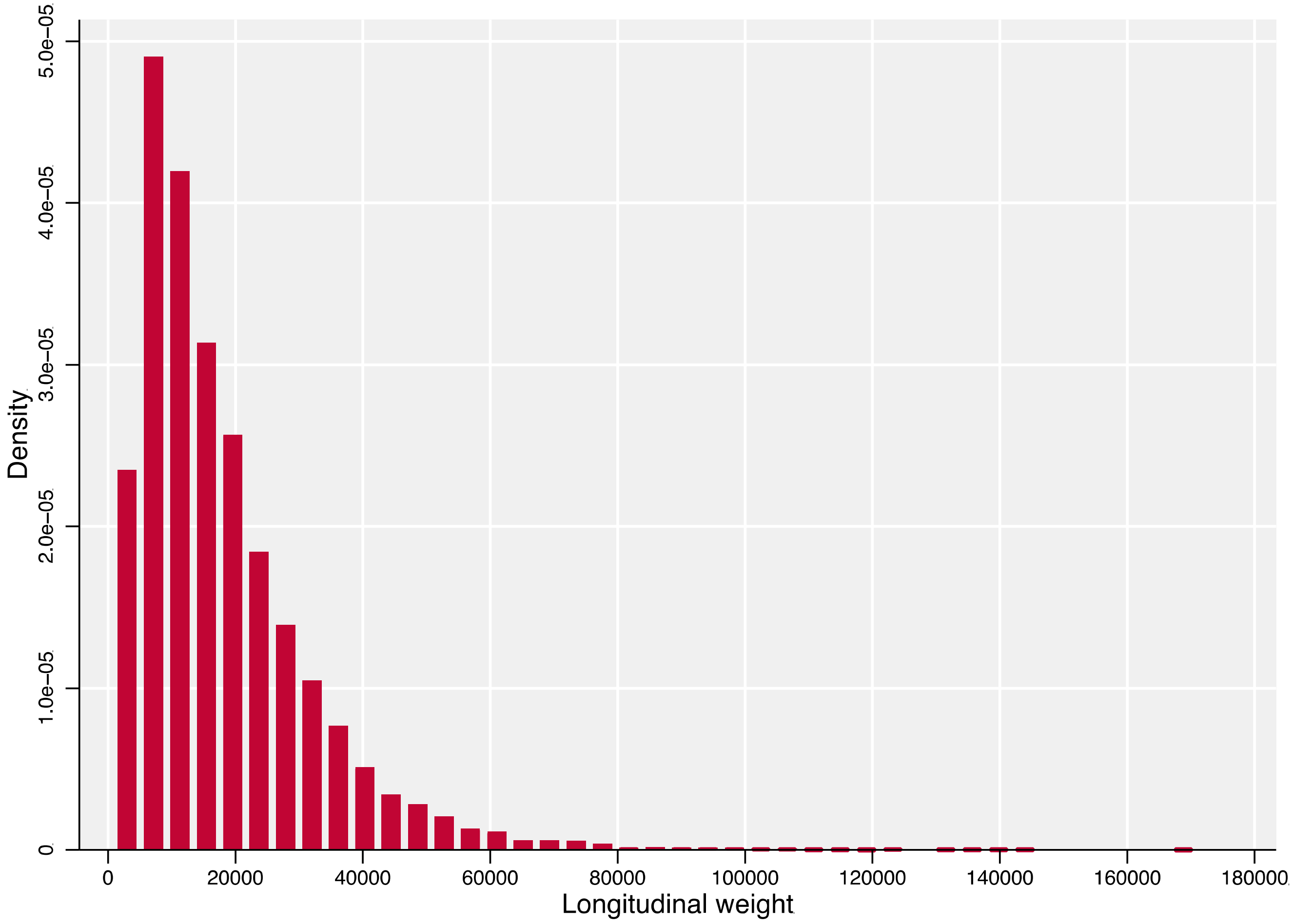

© 2021 Unger ES et al. *JAMA Health Forum.*

eReferences

1. Agency for Healthcare Research and Quality Center for Financing, Access, and Cost Trends. *MEPS HC-197H: 2017 Home Health Visits Documentation*.; 2019. https://www.meps.ahrq.gov/mepsweb/data_stats/download_data_files_detail.jsp?cboPufNumber=HC-197H

2. Cameron AC, Miller DL. A practitioner's guide to cluster-robust inference. *J Hum Resour*. 50(2):317-372.

3. Fleishman JA, Zuvekas SH. Global self-rated mental health: associations with other mental health measures and with role functioning. *Med Care*. 2007;45(7):602-609. doi:10.1097/mlr.0b013e31803bb4b0

© 2021 Unger ES et al. *JAMA Health Forum.*
